# Variability of control data and relevance of observed group differences in five oral toxicity studies with genetically modified maize MON810 in rats

**DOI:** 10.1007/s00204-016-1857-x

**Published:** 2016-10-11

**Authors:** Kerstin Schmidt, Jörg Schmidtke, Paul Schmidt, Christian Kohl, Ralf Wilhelm, Joachim Schiemann, Hilko van der Voet, Pablo Steinberg

**Affiliations:** 1 BioMath GmbH, Schnickmannstr. 4, 18055 Rostock, Germany; 2 Institute for Biosafety in Plant Biotechnology, Julius Kühn Institute, Federal Research Centre for Cultivated Plants, Erwin-Baur-Str. 27, 06484 Quedlinburg, Germany; 3 Wageningen UR Biometris, Postbus 16, 6700 AA Wageningen, The Netherlands; 4 Institute for Food Toxicology and Analytical Chemistry, University of Veterinary Medicine Hannover, Bischofsholer Damm 15, 30173 Hannover, Germany; 5 University of Hohenheim, Biostatistics (340c), Fruwirthstr. 23, 70599 Stuttgart, Germany

**Keywords:** 90-day toxicity study, Genetically modified plants, Maize, Risk assessment, Historical control data, Linear mixed models, Standardized effect sizes, Statistical power, Consistency

## Abstract

The data of four 90-day feeding trials and a 1-year feeding trial with the genetically modified (GM) maize MON810 in Wistar Han RCC rats performed in the frame of EU–funded project GRACE were analysed. Firstly, the data obtained from the groups having been fed the non–GM maize diets were combined to establish a historical control data set for Wistar Han RCC rats at the animal housing facility (Slovak Medical University, Bratislava, Slovakia). The variability of all parameters is described, and the reference values and ranges have been derived. Secondly, the consistency of statistically significant differences found in the five studies was analysed. In order to do so, the body weight development, organ weight, haematology and clinical biochemistry data were compared between the studies. Based on the historical control data, equivalence ranges for these parameters were defined, and the values measured in the GM maize–fed groups were compared with these equivalence ranges. Thirdly, the (statistical) power of these feeding studies with whole food/feed was assessed and detectable toxicologically relevant group differences were derived. Linear mixed models (LMM) were applied, and standardized effect sizes (SES) were calculated in order to compare different parameters as well as to provide an overall picture of group and study differences at a glance. The comparison of the five feeding trials showed a clear study effect in the control data. It also showed inconsistency both in the frequency of statistically significant differences and in the difference values between control and test groups.

**Electronic supplementary material:**

The online version of this article (doi:10.1007/s00204-016-1857-x) contains supplementary material, which is available to authorized users.

## Introduction

### Feeding studies with whole food/feed to assess the safety of genetically modified crops—the EU−funded project GRACE

The safety of GM plants is a subject of intense political and societal debate, characterized by diverging positions in different EU Member States. For example, there is an ongoing debate regarding the necessity of 90-day animal feeding studies to assess the safety of genetically modified (GM) plants. In 2013, a new Commission Implementing Regulation (European Commission 2013) came into force, which made 90-day feeding studies with whole food/feed mandatory in the frame of the safety assessment process of GM plants and derived products. This move was the result of a long-lasting discussion between the Member States and the European Commission aiming to incorporate the European Food Safety Authority (EFSA) Guidance for the GMO risk assessment into a legal text.

Before the Implementing Regulation became effective, 90-day animal feeding studies were only requested by EFSA in indicated cases (EFSA 2011a). Given the now mandatory but untargeted nature of the 90-day feeding trials, the challenge was to determine the scientific value and limitations of such studies and how they should be interpreted within the risk assessment process. In this context, the EU-funded project GRACE comparatively evaluated the use of 90-day animal feeding trials, animal studies with an extended time frame as well as analytical, in vitro and in silico studies on GM maize in the GMO risk assessment process.

The performance of subchronic toxicity studies in form of 90-day feeding trials and of chronic toxicity studies in form of 1-year feeding trials using whole food or feed as test material is challenging. This is due to the fact that the available internationally accepted test guidelines such as the OECD Test Guidelines 408 (OECD 1998) and 452 (OECD 2009) were originally developed to test the potential toxicity of chemicals.

There is a big difference between whole food or feed and chemicals as test material for this kind of studies. Chemicals can be administered at dose levels that are much higher than those to which human beings are exposed. Such a testing approach is not possible with whole food or feed, since the incorporation of high amounts of a crop to whole food or feed might lead to a nutritional imbalance. Therefore, when wanting to test the safety of GM crops, the OECD Test Guidelines used to test the potential toxicity of chemicals need to be adapted to meet the constraints of whole food or feed testing.

Therefore, EFSA developed a guidance document/recommendations for the performance of feeding studies on whole food and feed in rodents based on the OECD Test Guidelines 408 and 453 (EFSA 2011b, 2013, 2014), but these documents do not provide prescriptive test protocols to carry out such experiments. Hence, four 90-day feeding trials and a 1-year feeding trial with whole GM and non-GM plant material to validate and refine the suggested approaches were performed in the frame of the GRACE project (see below).

### Specific challenges of feeding trials with whole food/feed

From the point of view of the frequency with which parameters are assessed, two types of parameters are analysed in feeding studies. On the one hand, body weight and feed consumption are recorded once per week (“weight and feed consumption data”). On the other hand, organ weights, haematology, clinical biochemistry, urinalysis as well as gross necropsy and histopathology parameters are determined once at the end of the study (“other parameters”). All these parameters are compared between the groups and tested with respect to relevant baseline values to identify any test substance– and dose–dependent toxic responses (Schmidt et al. 2016).

The first critical issue to be pointed out is that the different parameters are measured in different scales and in different units. The toxicological relevance of deviations differs from parameter to parameter. Nevertheless, for obvious reasons, all parameters are measured for the same fixed number of animals, as proposed, e.g. by OECD and EFSA. This approach results in a broad range of (possibly insufficient) statistical power values.

The second issue is that historical data (from the respective lab) are very important when assessing the toxicological relevance of group differences measured in a feeding study. This is due to the fact that a natural variation among rats of an outbred strain such as the Wistar Han RCC strain used in all feeding trials performed in the course of the GRACE project does in fact exist. As stated, for example, in the OECD Test Guideline 452 (OECD 2009), historical control data may be valuable in the interpretation of the results of the study, e.g. in the case when there are indications that the data provided by the concurrent controls are substantially out of line when compared to recent data from control animals from the same test facility/colony. Historical control data, if evaluated, should be submitted from the same laboratory and relate to animals of the same age and strain generated during the 5 years preceding the study in question (OECD 2009). Unfortunately, no historical data regarding feeding trials with diets containing up to 33 % maize were available at the animal housing facility of the Slovak Medical University in Bratislava, Slovakia, in which all feeding trials performed in the frame of the GRACE project were run. Consequently, minimum differences of toxicological relevance could not be set for a prospective power analysis.

The third issue is related to the limited possibility of adding the GM crop to the feed at an inclusion rate that eventually will induce signs of toxicity and, thus, will allow to observe a dose–response relationship. In the frame of toxicity studies, chemicals are usually administered at dose levels that are much higher than the probable human exposure levels, thereby leading to toxicologically relevant results in the high-dose groups. Such an approach is not always possible with whole food or feed, since high inclusion rates might result in nutritional imbalanced diets (EFSA 2011b). EFSA (2014) suggested a 50 % incorporation rate as a reference value for a high dose of genetically modified maize in 90-day feeding trials in rodents, based on a study by Zhu et al. (2013) in Sprague–Dawley rats fed the glyphosate-resistant *G2*-*aroA* maize. At the present time, it remains unknown whether an incorporation rate of 50 % maize will lead to a nutritional imbalance in long-term feeding trials with a duration ≥1 year.

To handle the issue of statistical power in the case of whole food/feed studies, EFSA proposed to base the sample size on a pre-specified effect size defined in standard deviation (SD) units, thereby providing an example in which, based on data from previous toxicity tests and for all parameters, a difference of one SD or less is of little toxicological relevance (EFSA 2011b). EFSA also recommends the use of fewer dose levels but more animals in the control and high-dose groups to maximize the power of the study. To handle the issue of toxicological relevance and to identify the range of the natural (non-positive) variability of parameters, EFSA recommends to consider historical background data available in the actual testing facility and—if such data is not available—to include further reference groups.

Two different MON810 varieties with their corresponding near–isogenic controls and four conventional maize varieties were used as plant material in the studies described here. The test approaches comprised the following four 90-day feeding trials as well as a 1-year feeding trial:

- Study A: 90-day study with Monsanto MON810 maize
- Study B: 90-day study with Pioneer MON810 maize
- Study C: 1-year study with Monsanto MON810 maize
- Study D: Longitudinal and metabolomics 90-day study with Monsanto MON810 maize
- Study E: Longitudinal and metabolomics 90-day study with Pioneer MON810 maize.

The rat feeding trials A, B and C were conducted by taking into account the EFSA Guidance on conducting repeated−dose 90-day oral toxicity study in rodents on whole food/feed (EFSA 2011b) and the OECD Test Guidelines 408 and 452 (OECD 1998, 2009), and the results of these feeding trials have recently been published (Zeljenková et al. 2014, 2016). The longitudinal and metabolomics 90-day studies, as they were originally named by the GRACE consortium, refer to 90-day feeding trials performed in the same way as were the studies A and B with one exception: In the studies D and E, blood and urine were collected from the tail vein of the animals at day 7 and after 1, 2 and 3 months for immunological and metabolomics analyses, while in the studies A and B blood (no urine) was collected once at the end of the feeding trial.

### Aims of the present study

In a first step, the data sets obtained in the feeding trials D and E, including the daily clinical observations, the ophthalmological findings, the body weight of the animals, the relative organ weights, the haematology and clinical biochemistry parameters as well as the gross necropsy and histological findings, are reported and discussed.

In a second step, the data sets of the feeding trials D and E together with those of the recently published studies A, B and C (Zeljenková et al. 2014, 2016) were used to address the specific issues described above. Specifically,

- “historical” control data of Wistar Han RCC rats fed whole food/feed diets containing 33 % non-GM maize for the animal housing facility at the Slovak Medical University (Bratislava, Slovakia) were compiled;
- the homogeneity of the historical control data was assessed;
- the toxicological relevance of any observed statistically significant differences between the control and test groups was analysed by assessing the consistency of such differences between GM and non-GM diet groups across the five studies and by comparing the data obtained with the GM-containing diets to the historical data;
- the (statistical) power of feeding studies with whole food/feed to detect toxicologically relevant group differences was assessed.

## Materials and methods

Data from five 90-day feeding trials (studies A–E) performed in the frame of the GRACE project were available (Table 1). A GM maize variety with the MON810 event from Monsanto (St. Louis, MO, USA) was used in the studies A, C and D, while a GM maize variety with the MON810 event from DuPont Pioneer Hi-Bred International (Johnston, IA, USA) was used in the studies B and E (Table 1). The European Commission requested the GRACE consortium to use the MON810 maize for the feeding trials, and the varieties used in the feeding trials are those being normally cultivated in Catalonia, Spain. In all studies, at least two GM groups (rats fed diets containing 11 and 33 % GM maize) and a control group (rats fed a diet containing 33 % non-GM maize) were included. Group sizes were chosen to be 16 per sex in studies A and B according to an example in the EFSA Guidance Document (EFSA 2011b), 20 per sex in study C according to the OECD Test Guideline 452 (OECD 2009), and 10 per sex in studies E and D according to the OECD Test Guideline 408 (OECD 1998), see Table 1. The data of the feeding trials A and B were analysed by Zeljenková et al. (2014; for details see Schmidt and Schmidtke 2014), those of the feeding trial C by Zeljenková et al. (2016; for details see Schmidt et al. 2015a) and those of the feeding trials D and E in this report (for details also see Schmidt et al. 2015b). The design of the 90-day studies A, B, D and E was the same with one exception: Blood and urine were collected at day 7 and after 1, 2 and 3 months for immunological and metabolomics analyses in the studies D and E, while blood, but no urine, was collected once at the end of the feeding trials A and B. For all trials, data quality and distribution checks and thereafter the above-mentioned individual analyses were performed (as described in Schmidt et al. 2016). Raw data and statistical analysis reports of all studies are available at the CADIMA website. The open-access database CADIMA is a non-profit internet portal aiming to increase the transparency and traceability of information being associated with the impact/risk assessment of plant genetic improvement technologies. Among others, it grants access to raw data generated by associated research activities (see www.cadima.info).

**Table 1:** Overview of the analysed feeding studies and the used maize varieties

Study	Groups					No. of animals		ExpU* per group and sex	GMO variety owner	Study type
	Control	11 % GMO	33 % GMO	33 % conv. 1	33 % conv. 2	Per group and sex	Total			
A	DKC6666	DKC6667-YG	DKC6667-YG	PR33W82	SY-NEPAL	16	160	8	Monsanto	90-day oral subchronic
B	PR32T16	PR33D48	PR33D48	PR32T83	DKC6815	16	160	8	Pioneer	90-day oral subchronic
C	DKC6666	DKC6667-YG	DKC6667-YG		SY-NEPAL	20	160	10	Monsanto	1-year oral chronic
D	DKC6666	DKC6667-YG	DKC6667-YG			10	60	5	Monsanto	90-day longitudinal/metabolomics
E	PR32T16	PR33D48	PR33D48			10	60	5	Pioneer	90-day longitudinal/metabolomics

* ExpU = Experimental Unit. Two rats were housed per cage, the experimental unit being the cage

### Maize varieties and diets

Studies A and B used maize material harvested in the 2012 season in Pla de Foixa (Girona, Catalonia, Spain, 42°05′N, 3°E). Studies D and E used the same batch of diets as studies A and B, respectively, but the diets were stored further on at −21 °C for 10 months. Study C used maize material from the 2013 harvest from the same region, but from different sites than the other studies. The maize varieties and the diets used are listed in Table 1.

### Data structure

In all trials, the two treatment groups (11 % GM maize [11 % GMO] and 33 % GM maize [33 % GMO]) were compared to a control group (33 % non-GM control maize, [control]). Furthermore, additional groups fed conventional maize varieties were incorporated in trial A ([conventional1] and [conventional2]), in trial B ([conventional3] and [conventional4]) and in trial C (again [conventional2]).

*Body weight and feed consumption data*: Each rat was weighed 14 times—starting on the first day of the trial and then proceeding on a weekly basis until the last weighing 13 weeks later. Feed consumption was also determined once per week and reported as the total amount of feed consumed by two animals in one cage per week.

**Fig. 1:**
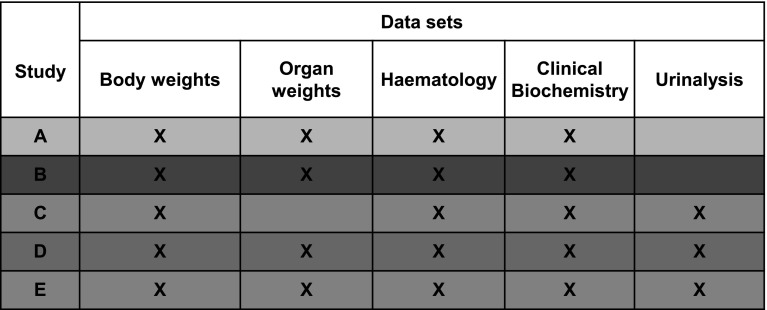
Available data sets of the five feeding trials at *t* = 13 weeks. Study colours (A = *blue*, B = *red*, C = *green*, D = *brown*, E = *purple*) are applied in all graphs (colour figure online)

*All other parameters*: The following haematology parameters were determined: the white blood cell count (WBC), red blood cell count (RBC), haemoglobin concentration (HGB), haematocrit (HCT), mean cell volume (MCV), mean corpuscular haemoglobin (MCH), mean corpuscular haemoglobin concentration (MCHC), platelet count (PLT) and lymphocyte count (LYM) as well as the differential leukocyte count. The following clinical biochemistry parameters were measured: alkaline phosphatase (ALP), alanine aminotransferase (ALT), aspartate aminotransferase (AST), albumin (ALB), total protein (TP), glucose (GLU), creatinine (CREA), urea (U), cholesterol (CHOL), triglycerides (TRG), calcium (Ca), chloride (Cl), potassium (K), sodium (Na) and phosphorus (P). Moreover, except for trial C (since this was a 1-year feeding trial, rats were not sacrificed at t = 90 days), the weight of the kidneys, spleen, liver, adrenal glands, pancreas, lung, heart, thymus, testes, epididymides, uterus, ovaries and brain was recorded (the collated primary data are available on the website http://www.cadima.info). An overview of the data sets determined in all five studies is shown in Fig. 1.

In all five feeding trials, two animals per cage were held. According to the EFSA Guidance Document (EFSA 2011b), the cage was defined as the experimental unit and means per cage were calculated for all parameters prior to the statistical analysis. All animals survived until *t* = 90 days, i.e. there were no missing data.

It has to be pointed out that statistical analyses were only performed on the quantitative parameters, i.e. the body weight, haematology, clinical biochemistry and relative organ weight data. Clinical findings were only, if at all, sporadically observed and, therefore, did not undergo any sort of statistical analysis (for details see Schmidt and Schmidtke 2014; Zeljenková et al. 2014, 2016; Schmidt et al. 2015a, b).

### Individual study analyses

For each individual study, body weight and feed consumption data were analysed by applying mixed models, separately for each gender, and by using the restricted maximum likelihood (REML) algorithm with Toeplitz/AR(1) covariance structure. This approach considers the changes in both repeatedly measured parameters over all points in time and is a much more generalized model than nonlinear models or growth curves (Schmidt et al. 2016). The group (five/four/three levels) was considered a fixed factor. The factor week (time in weeks from the start of the experiment) or day (time in days from the start of the experiment) was considered a quantitative fixed factor (repeated measurements). For the resulting least square means, standardized effect sizes with their 95 % confidence intervals were calculated for the comparisons of particular interest (mainly 11 % GMO–control and 33 % GMO–control, in some studies also conventional–control) according to Nakagawa and Cuthill (2007). Moreover, a “classical” analysis of variance (ANOVA) was carried out, separately for each gender and each week. For the comparisons of particular interest (11 % GMO–control, 33 % GMO–control and conventional–control), post hoc Dunnett’s tests were performed after the ANOVA.

**Fig. 2:**
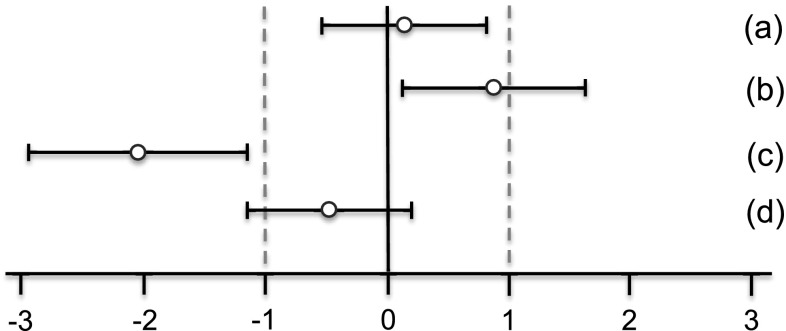
Simplified version of a graph allowing the visual assessment of statistical significance and toxicological relevance of group comparisons. The SES point estimate (*circle*) and the 95 % confidence limits (*whiskers, bars* showing confidence interval) illustrate the (standardized) effect size between two groups. The *vertical black line* indicates no statistically significant difference (zero difference), *vertical dashed grey lines* indicate toxicological relevance limits (here 1.0 SD, according to the study design). If the confidence interval bars cross the *zero line* but not the *grey dashed lines*, i.e. they lie within the ±1.0 SD limits, there is evidence for no statistical significance as well as no toxicological relevance (case a). Two groups are significantly different when the *confidence interval bars* do not cross the *black vertical line* (cases b, c). The effect size between two groups is supposed to be toxicologically relevant, when the *confidence interval bars *lie outside the ±1.0 limits (case c). Case b indicates statistical significance, but no clear toxicological relevance. Case d indicates no statistical significance, but no clear negation of toxicological relevance (reproduced from Zeljenková et al. 2014)

In the case of all other parameters, standardized effect sizes as well as their 95 % confidence intervals were calculated for the same group pairs and separately for each gender (Festing 2014; Schmidt et al. 2016). All standardized effect size (SES) estimates are graphically shown, thereby displaying both statistical significance and presumptive decision thresholds for the toxicological assessment of each of the parameter comparison results (Fig. 2). Setting equivalence limits based on toxicological relevance is not easy and the subject of continued debate. In this paper, a working value for toxicological relevance is pragmatically set at equivalence limits of ±1.0 SD, as previously used in a simple example by EFSA (EFSA 2011b). It should therefore be kept in mind that future decisions on relevant equivalence limits may influence the equivalence results as presented in the current paper. The body weight as well as all other parameters are shown in the same graph (separately for male and female rats), thereby forming an overall pattern and allowing the assessment of group comparisons at a glance. Again, an additional “classical” analysis with an ANOVA/post hoc Dunnett’s tests or nonparametric Kruskal–Wallis/Wilcoxon tests was carried out for each gender.

### Compilation of historical control data from the groups fed 33 % non-GM maize-containing diets

In the five feeding trials, two control and four other conventional diets (all together named hereafter “non-GM diets”) were used (Table 2). The parameters measured in these groups were combined to compile historical non-GM control data (point and interval estimates) for Wistar Han RCC rats fed a diet containing 33 % conventional maize in the animal housing facility at the Slovak Medical University (Bratislava, Slovakia). Reference statistics (mean, SD, minimum, maximum, percentiles: median, 5 and 95 % percentiles) were calculated for the weight and laboratory parameters from all non-GM groups in the five feeding trials. To describe the variability, two measures were used: the coefficient of variation (relative SD, *i.e.* ratio of the SD to the mean) and the relative width of the 90 % central interpercentile range (5–95 % percentile) to the mean.

**Table 2:** Number of values (cages) available in non-GM groups for the calculation of the historical control data ranges (78 values available for both male and female rats)

Study	Non–GM varieties						
	DKC6666	DKC6815	PR32T16	PR33W82	PR32T83	SY–NEPAL	Per study and sex
A	8			8		8	24
B		8	8		8		24
C	10					10	20
D	5						5
E			5				5
Per variety and sex	23	8	13	8	8	18	78

The distribution of the non-GM diet-related data was graphically displayed in boxplots, separately for male and female animals and showing the median, the interquartile range = box length and extreme cases of individual variables in two categories, namely values between 1.5 and 3 box lengths from the upper or lower edge of the box and cases with values more than 3 box lengths from the upper or lower edge of the box (shown in the statistical analysis reports: Schmidt and Schmidtke 2014; Schmidt et al. 2015a, b). All calculations and graphs were based on the values per cage (experimental unit).

### Assessment of the homogeneity of the historical control data set

Firstly, to evaluate the reproducibility of the parameter measurements, an ANOVA was applied to compare the means of the individual studies. Secondly, to evaluate the magnitude of study–specific influences on the variance of the historical data set, a principal component analysis (PCA) followed by a cluster analysis (CA) was applied. The PCA was applied to combined male–female data to assess its suitability to separate expected gender differences (e.g. in the body and organ weights). The corresponding values were converted into two principal components, groupwise for body weight, haematology, clinical biochemistry and organ weight. Then, these components were grouped into five clusters. The number of components and clusters were chosen both for practical and technical reasons. For PCA, varimax rotation with Kaiser normalization was used, and a hierarchical cluster analysis was applied to form clusters based on their quadratic Euclidian distance. The resulting components were displayed graphically in a scatter plot.

### Evaluation of the toxicological relevance of statistically significant differences

In its Scientific Opinion on statistical significance and biological relevance (EFSA 2011c), EFSA defined “The objective of carrying out an empirical study is usually to identify the existence of relevant biological effects at the population level using statistical tools to detect them. Therefore, the identification of statistical significance is only part of the evaluation of the biological relevance.”

As a basic principle, in each study, it was evaluated whether the observed statistically significant differences between the control and the test groups were in line with an effect pattern indicative of potential toxicity (e.g. liver toxicity).

To support the evaluation of the toxicological relevance of statistically significant differences identified in all five studies, the following approach was applied:

(i) a consistency check of differences across studies. A statistically significant difference may be considered “consistent” and might be an indicator of a toxicologically relevant (i.e. adverse) alteration if it is reproduced across studies.
(ii) a comparison of the values observed in the GM maize-fed groups with the historical control data set compiled from the non-GM groups of the five studies. A value outside of the historical control data range might be an indicator of a toxicologically relevant (i.e. adverse) alteration.

#### Consistency of statistically significant differences between GM and non-GM diet groups

Firstly, in order to visually evaluate the consistency of the statistically significant differences, weight development curves as well as SES graphs of all five studies were combined in one graph, allowing a direct visual comparison of differences observed in the individual studies.

Secondly, to evaluate the consistency of the frequencies of statistically significant differences, total and relative numbers of such differences observed in haematology, clinical biochemistry and organ weight measurements of all five feeding trials were compared.

Thirdly, to evaluate the consistency of the difference values, the absolute differences between GM and non-GM diet groups were listed in form of tables for all studies, separately for male and female animals. Statistically significant differences (positive or negative) were highlighted to easily compare their absolute values and their incidence.

#### Comparison of the values observed in the GM maize-fed groups with the historical control data

The historical control data compiled from the non-GM groups of the five studies (Table 3) were used to set up simplified equivalence ranges (baselines) on the basis of the 1-SD approach. The mean and SD calculated for all non-GM (control and conventional) groups of the five studies were taken to set a lower equivalence limit (=mean–SD) and an upper equivalence limit (=mean + SD). Thereafter, it was checked whether the means of the individual parameters measured in the 11 % GMO- and 33 % GMO-fed groups fell within or outside these limits.

**Table 3:** Compilation of historical control data for male (A) and female (B) rats fed 33 % non−GM maize−containing diets in the GRACE project 90-day feeding trials A, B, C, D and E

Parameter	Count	Mean^a^	Std Deviation^a^	Median^a^	Minimum^a^	Maximum^a^	Percentile 05^a^	Percentile 95^a^
A: Male rats								
*Body weight*								
Week 0 [g]	78	149.9	6.5	150.2	137.6	167.8	138.9	161.5
Week 1 [g]	78	198.1	7.8	197.3	180.9	217.1	186.2	213.3
Week 2 [g]	78	240.1	9.1	239.3	218.9	262.0	225.6	258.6
Week 3 [g]	78	276.4	11.9	276.3	249.3	303.5	256.2	297.1
Week 4 [g]	78	303.5	14.9	303.3	265.3	335.8	280.3	330.2
Week 5 [g]	78	328.4	15.4	327.3	286.2	360.5	305.6	357.3
Week 6 [g]	78	348.4	17.1	346.7	304.0	384.7	322.1	379.1
Week 7 [g]	78	364.6	18.4	362.6	320.8	403.3	334.8	397.5
Week 8 [g]	78	377.6	20.4	377.1	326.7	422.3	347.0	411.8
Week 9 [g]	78	391.1	20.7	390.5	344.7	436.0	357.0	424.9
Week 10 [g]	78	401.3	21.2	401.1	356.4	451.4	366.5	437.7
Week 11 [g]	78	411.9	22.7	412.3	363.6	466.8	378.0	453.3
Week 12 [g]	78	419.7	22.4	416.6	372.1	474.8	387.2	465.2
Week 13 [g]	78	422.9	23.5	417.8	377.2	488.5	388.9	465.9
*Haematology*								
WBC [10^3^/µl]	78	10.14	2.88	9.28	5.10	18.55	6.21	15.42
RBC [10^6^/µl]	78	8.30	0.40	8.35	6.88	9.11	7.46	8.84
HGB [g/dl]	78	16.09	0.83	16.20	12.00	17.25	14.23	16.96
HCT [%]	78	45.98	2.25	46.13	38.10	49.30	41.24	48.87
MCV [fl]	78	55.40	1.04	55.30	52.85	58.45	54.00	57.13
MCH [pg]	78	19.40	0.62	19.40	16.45	20.50	18.37	20.33
MCHC [g/dl]	78	35.02	0.97	34.95	29.30	36.50	33.94	36.31
PLT [10^3^/µl]	78	751.64	159.51	773.50	207.50	1041.50	369.98	933.45
LYM [10^3^/µl]	78	8.05	2.43	7.80	3.60	12.70	4.12	11.83
Lymphocytes [%]	78	77.31	5.05	78.63	60.50	85.25	66.68	84.41
Neutrophils [%]	78	17.51	4.91	16.00	8.75	31.00	11.50	28.39
Monocytes [%]	78	3.47	1.34	3.50	1.00	9.50	1.86	5.75
Eosinophils [%]	78	1.69	0.79	1.50	0.50	3.50	0.61	3.25
Basophils [%]	78	0.00	0.00	0.00	0.00	0.00	0.00	0.00
*Clinical biochemistry*								
ALP [µkat/l]	78	1.35	0.26	1.29	0.92	2.10	1.00	1.81
ALT [µkat/l]	78	0.63	0.67	0.53	0.31	6.00	0.41	0.74
AST [µkat/l]	78	1.44	0.79	1.19	0.78	6.20	0.83	2.50
ALB [g/l]	78	34.35	3.17	33.68	27.80	41.55	29.84	39.82
GLU [mmol/l]	78	8.63	2.14	8.57	4.90	13.75	5.27	12.68
CREA [µmol/l]	78	43.31	6.61	42.93	29.80	58.35	32.95	55.98
TP [g/l]	78	60.96	2.80	61.03	55.50	68.30	56.56	65.30
U [mmol/l]	78	6.11	0.75	6.03	4.92	8.71	4.95	7.41
CHOL [mmol/l]	78	2.29	0.29	2.28	1.78	3.20	1.86	2.91
Ca [mmol/l]	78	2.57	0.20	2.54	2.15	3.03	2.18	2.95
Cl [mmol/l]	78	108.03	6.64	107.00	100.00	150.00	102.23	116.55
K (mmol/l)	78	4.83	0.57	4.78	3.85	7.20	4.02	5.81
Na [mmol/l]	78	148.97	9.94	147.25	138.00	215.00	140.00	163.85
P [mmol/l]	78	2.60	0.52	2.53	1.76	5.26	2.01	3.19
TRG [mmol/l]	78	0.72	0.24	0.71	0.29	1.38	0.33	1.19
*Organ weights*								
Kidney (right) [%]	78	0.283	0.018	0.285	0.239	0.327	0.248	0.310
Kidney (left) [%]	78	0.283	0.017	0.285	0.244	0.325	0.250	0.309
Spleen [%]	78	0.185	0.017	0.184	0.155	0.228	0.159	0.220
Liver [%]	78	2.214	0.169	2.180	2.026	2.968	2.037	2.581
Adrenal gland (right) [%]	78	0.006	0.001	0.006	0.004	0.008	0.004	0.008
Adrenal gland (left) [%]	78	0.007	0.001	0.006	0.005	0.012	0.006	0.008
Lung [%]	78	0.330	0.033	0.326	0.270	0.434	0.286	0.388
Heart [%]	78	0.230	0.012	0.228	0.208	0.257	0.214	0.257
Thymus [%]	78	0.100	0.019	0.097	0.053	0.137	0.072	0.135
Pancreas [%]	78	0.134	0.020	0.136	0.090	0.175	0.102	0.167
Testis (right) [%]	78	0.468	0.037	0.465	0.403	0.566	0.411	0.549
Testis (left) [%]	78	0.468	0.035	0.475	0.400	0.557	0.404	0.535
Epididymis (right) [%]	78	0.152	0.012	0.152	0.129	0.179	0.132	0.176
Epididymis (left) [%]	78	0.154	0.012	0.155	0.131	0.178	0.134	0.175
Brain [%]	78	0.529	0.027	0.530	0.461	0.587	0.480	0.573
B: Female rats								
*Body weight*								
Week 0 [g]	78	132.4	6.4	131.7	119.9	148.3	123.7	145.4
Week 1 [g]	78	156.9	7.1	156.4	139.5	173.7	145.7	168.9
Week 2 [g]	78	176.7	8.7	176.0	157.6	205.7	161.3	191.7
Week 3 [g]	78	192.1	10.8	191.5	157.3	221.2	177.8	211.8
Week 4 [g]	78	203.1	11.7	201.7	169.1	238.9	186.2	225.0
Week 5 [g]	78	214.5	12.2	213.5	193.5	253.7	195.8	237.4
Week 6 [g]	78	222.8	13.2	220.8	196.3	263.4	204.3	244.6
Week 7 [g]	78	229.7	14.0	227.9	199.2	269.5	210.0	254.4
Week 8 [g]	78	235.4	14.2	233.9	205.6	277.1	214.8	261.9
Week 9 [g]	78	239.0	14.9	237.2	209.7	284.9	217.5	268.0
Week 10 [g]	78	244.1	15.8	242.5	208.4	288.3	222.7	274.1
Week 11 [g]	78	249.4	16.4	247.7	213.9	295.2	227.0	282.3
Week 12 [g]	78	251.7	17.6	249.3	219.1	300.9	226.7	284.1
Week 13 [g]	78	253.4	18.4	249.9	214.6	305.3	228.1	290.0
*Haematology*								
WBC [10^3^/µl]	78	7.84	2.07	8.00	3.00	15.90	4.34	10.76
RBC [10^6^/µl]	78	7.67	0.29	7.64	7.15	8.38	7.20	8.29
HGB [g/dl]	78	15.45	0.47	15.45	13.60	16.50	14.70	16.23
HCT [%]	78	43.83	1.28	43.75	41.00	47.10	41.62	46.23
MCV [fl]	78	57.19	1.15	57.30	54.40	60.00	55.04	59.35
MCH [pg]	78	20.06	1.31	20.20	10.75	22.30	19.17	21.22
MCHC [g/dl]	78	35.05	2.09	35.20	18.95	38.50	34.12	36.28
PLT [10^3^/µl]	78	783.61	135.85	787.75	294.00	998.50	487.55	989.55
LYM [10^3^/µl]	78	5.99	1.84	5.70	2.10	13.30	3.05	8.91
Lymphocytes [%]	78	78.07	6.15	79.50	62.25	86.25	63.61	85.25
Neutrophils [%]	78	17.56	5.75	16.38	8.50	33.50	10.34	30.69
Monocytes [%]	78	2.68	0.86	2.75	1.00	5.25	1.50	4.66
Eosinophils [%]	78	1.68	0.76	1.63	0.00	4.50	0.61	3.25
Basophils [%]	78	0.04	0.09	0.00	0.00	0.25	0.00	0.25
*Clinical biochemistry*								
ALP [µkat/l]	78	0.59	0.10	0.58	0.33	0.85	0.43	0.76
ALT [µkat/l]	78	0.48	0.14	0.47	0.19	1.13	0.26	0.69
AST [µkat/l]	78	1.46	0.84	1.03	0.75	4.12	0.79	3.33
ALB [g/l]	78	42.75	4.38	42.58	32.90	51.65	35.76	49.88
GLU [mmol/l]	78	6.82	1.29	6.60	3.52	9.81	4.95	9.06
CREA [µmol/l]	78	42.67	7.40	40.80	29.50	62.00	34.28	59.66
TP [g/l]	78	68.62	5.47	69.38	56.45	80.45	57.42	76.64
U [mmol/l]	78	5.62	0.72	5.55	4.55	7.65	4.59	6.90
CHOL [mmol/l]	78	2.01	0.29	2.00	1.45	2.68	1.60	2.55
Ca [mmol/l]	78	2.59	0.23	2.59	1.88	3.00	2.14	2.95
Cl [mmol/l]	78	106.54	4.27	106.50	98.50	118.00	100.00	114.55
K (mmol/l)	78	4.42	0.82	4.20	3.50	8.65	3.67	6.26
Na [mmol/l]	78	148.16	5.37	148.00	137.50	161.00	139.00	156.30
P [mmol/l]	78	2.33	0.41	2.38	1.55	3.60	1.62	2.94
TRG [mmol/l]	78	0.52	0.16	0.53	0.16	0.99	0.27	0.75
*Organ weights*								
Kidney (right) [%]	78	0.316	0.019	0.314	0.278	0.358	0.284	0.350
Kidney (left) [%]	78	0.316	0.020	0.315	0.265	0.366	0.276	0.349
Spleen [%]	78	0.246	0.028	0.246	0.182	0.316	0.194	0.295
Liver [%]	78	2.508	0.196	2.513	2.197	3.289	2.250	2.869
Adrenal gland (right) [%]	78	0.014	0.002	0.014	0.008	0.019	0.010	0.017
Adrenal gland (left) [%]	78	0.015	0.002	0.015	0.011	0.019	0.012	0.018
Lung [%]	78	0.443	0.036	0.443	0.343	0.517	0.384	0.497
Heart [%]	78	0.281	0.019	0.279	0.244	0.320	0.250	0.318
Thymus [%]	78	0.131	0.018	0.129	0.100	0.174	0.109	0.165
Pancreas [%]	78	0.183	0.028	0.181	0.110	0.281	0.144	0.231
Uterus [%]	78	0.202	0.035	0.202	0.094	0.259	0.139	0.255
Ovary (right) [%]	78	0.025	0.005	0.026	0.016	0.039	0.017	0.031
Ovary (left) [%]	78	0.026	0.005	0.026	0.015	0.035	0.017	0.033
Brain [%]	78	0.835	0.052	0.839	0.705	0.957	0.735	0.924

^a^Derived from cage values (average for 2 rats)

### Assessment of the statistical power of the studies

The power or sensitivity of a statistical test (here: of a comparison of the results from a GM with a control group) is the probability that the test correctly identifies an actually existing effect. “Actually existing” refers to the effect of toxicological relevance that is defined in advance of the study and thus co-determines the sample size. EFSA (2011b) described an example in which differences of one SD unit were considered of little toxicological relevance, resulting in a sample size of 16 animals per group and sex (i.e. 8 cages). Consequently, feeding studies designed according to this example aim to detect group effects of one SD value for all parameters measured.

On the basis of the historical control data compiled from the non-GM groups of the five studies, in a first approach without factoring the study effect, absolute values of effect sizes detectable with a power of 0.8 or 0.9 and sample sizes of (i) 10 animals per group and sex (OECD 2009), (ii) 16 animals per group and sex (EFSA 2011b) or (iii) 20 animals per group and sex (OECD 1998) were determined for each single parameter. For these calculations, the experimental unit is the cage with two animals.

For all analyses and graphs, the SAS Software, version 9.4, from the SAS Institute Inc. (Cary, NC, USA) was used. SAS and all other SAS Institute Inc. product or service names are registered trademarks or trademarks of the SAS Institute Inc.

## Results

### 90-day feeding trials D and E

No signs of morbidity and mortality were observed throughout the 90-day feeding period, and the daily clinical observations did not reveal any signs of functional deficits in the feeding trials D and E. Furthermore, no ophthalmological alterations were visible in male and female rats fed the control, 11 % GMO and 33 % GMO diets in both trials. Body weight, relative organ weight as well as haematology and clinical biochemistry data were analysed as described in the “Materials and methods, Individual study analyses” section and are shown for study D in Table 1 of the Electronic Supplementary Material and for study E in Table 2 of the Electronic Supplementary Material. Moreover, all results (tables, graphs) are available in the Statistical Analysis Report at the CADIMA website www.cadima.info.

No significant differences in body weight development and feed consumption were observed between groups (in both studies, in male and female rats, and by using linear mixed model analysis).

For all the haematology and clinical biochemistry parameters, SES were calculated. In this context, a statistically significant difference means that the confidence interval of the SES to the control does not include the zero value, while “similar” means that the confidence interval of the SES to the control includes the zero value (Fig. 2).

The haematology parameters and the differential leukocyte counts were similar between the groups fed GM maize and the control groups of male and female rats in both trials with the exceptions, as described below. In trial D (Electronic Supplementary Material, Table 1), MCV, MCH and the percentage of eosinophils were significantly increased in male rats fed the 11 % GMO diet when compared to the control group, while the percentage of neutrophils was significantly decreased in male rats fed the 33 % GMO diet when compared to the control group. Furthermore, WBC were increased in female rats fed the 11 % GMO diet when compared to the control group, whereas WBC, MCHC and the number of lymphocytes per microlitre were significantly increased in female rats fed the 33 % GMO diet when compared to the control group. In trial E (Electronic Supplementary Material, Table 2), the percentage of neutrophils was significantly decreased in male rats fed the 11 % GMO diet, while the percentage of lymphocytes was significantly increased and the percentage of neutrophils was significantly decreased in male rats fed the 33 % GMO diet when compared to the control group. Moreover, in female rats fed the 11 % GMO diet, the percentage of lymphocytes was significantly decreased and the percentage of neutrophils was increased when compared to the control group, whereas WBC and the percentage of monocytes were decreased in the 33 % GMO group if compared to the control group.

Clinical biochemistry parameters in both trials were similar between the groups fed GM maize and the control groups of male and female rats with the exceptions, as described below. In trial D (Electronic Supplementary Material, Table 1), ALP activity was significantly decreased in male rats fed the 11 % GMO diet, and U was significantly decreased in the 33 % GMO group if compared to the corresponding control group. ALT, AST and U were significantly increased in female rats fed the 11 % GMO diet, while ALT, AST and TRG were significantly increased in female rats fed the 33 % GMO diet if compared to the corresponding control group. In trial E, no significant differences between rats being fed the control diet and those fed the GMO diets were observed.

The relative weight of all organs was similar between the groups fed GM maize, and the control groups of male and female rats except in trial D the weight of the left kidney were slightly lower in female rats fed the 11 % GMO diet than in the control rats (Electronic Supplementary Material, Table 1).

All parameters measured in the urine in both trials were similar with one exception: Osmolality was significantly increased in female rats fed the 33 % GMO diet when compared to the control group.

No signs of morbidity and mortality were observed throughout the 90-day feeding period, and the daily clinical observations did not reveal any signs of functional deficits in feeding trials D and E. No ophthalmological alterations were visible in male and female rats fed the control, 11 % GMO and 33 % GMO diets in both trials. No macroscopically visible alterations were observed in male and female rats fed the control, 11 % GMO and 33 % GMO diets in both trials with the exception of a male rat (No. 32) fed the 33 % GMO diet in trial E, in which the right seminal vesicle and coagulating gland had a reduced size but without accompanying histopathological alterations. A low number of histological changes were observed in the control and 33 % GMO groups in trials D and E and mostly were inflammatory reactions (Electronic Supplementary Material, Table 3). No pre-neoplastic and/or neoplastic lesions were observed. Since no treatment-related changes were observed between the control and high-dose groups, no further tissue analyses were carried out.

### Compilation of historical control data from groups fed 33 % non-GM maize-containing diets

The 90-day body weight, haematology, clinical biochemistry as well as relative organ weight data from all non-GM groups of the five studies (i.e. those having been fed a diet containing either a control, i.e. near-isogenic, non-GM, or another conventional maize variety) were transferred to a meta-data file. As in the case of all analyses of previous GRACE feeding trials, those values were excluded from the analysis that showed distinct haemolysis or that were outside the dynamic range of the analyser, but no other statistical outliers or extreme values were removed from the data set (see Schmidt and Schmidtke 2014; Schmidt et al. 2015a, b).

The compiled information on historical control values is shown in Table 3, in which the reference statistics (mean, median, SD, minimum, maximum, 5 and 95 % percentiles) for body weight per week, haematology and clinical biochemistry parameters as well as relative organ weights of all non-GM groups in the five studies are listed.

Means and medians of the non-GM groups for 93 % of the parameters differed by less than 5 %, thereby indicating symmetric distributions (Table 4, columns 2 and 5). Only a few extreme values were observed (not shown).

**Table 4:** Relative variability of historical control data: relative median−mean differences, coefficients of variation and relative widths of the 95 % central interpercentile range (rats fed diets containing 33 % non−GM maize, data based on cage means [2 rats/cage])

Parameter	Male			Female		
	Mean–median	Coeff. var.	90 % interval	Mean–median	Coeff. var.	90 % interval
*Body weight*						
Week 0 [g]	0.20 %	4.3 %	7.5 %	−0.52 %	4.8 %	8.2 %
Week 1 [g]	−0.40 %	3.9 %	6.8 %	−0.33 %	4.6 %	7.4 %
Week 2 [g]	−0.33 %	3.8 %	6.9 %	−0.43 %	4.9 %	8.6 %
Week 3 [g]	−0.04 %	4.3 %	7.4 %	−0.31 %	5.6 %	8.9 %
Week 4 [g]	−0.07 %	4.9 %	8.2 %	−0.70 %	5.8 %	9.6 %
Week 5 [g]	−0.33 %	4.7 %	7.9 %	−0.45 %	5.7 %	9.7 %
Week 6 [g]	−0.49 %	4.9 %	8.2 %	−0.91 %	5.9 %	9.1 %
Week 7 [g]	−0.55 %	5.0 %	8.6 %	−0.77 %	6.1 %	9.7 %
Week 8 [g]	−0.13 %	5.4 %	8.6 %	−0.64 %	6.0 %	10.0 %
Week 9 [g]	−0.15 %	5.3 %	8.7 %	−0.74 %	6.2 %	10.6 %
Week 10 [g]	−0.05 %	5.3 %	8.9 %	−0.67 %	6.5 %	10.5 %
Week 11 [g]	0.10 %	5.5 %	9.1 %	−0.68 %	6.6 %	11.1 %
Week 12 [g]	−0.74 %	5.3 %	9.3 %	−0.96 %	7.0 %	11.4 %
Week 13 [g]	−1.21 %	5.6 %	9.1 %	−1.36 %	7.3 %	12.2 %
*Haematology*						
WBC [10³/µl]	−8.54 %	28.4 %	45.4 %	2.00 %	26.4 %	40.9 %
RBC [10^6^/µl]	0.53 %	4.8 %	8.3 %	−0.42 %	3.8 %	7.1 %
HGB [g/dl]	0.68 %	5.1 %	8.5 %	0.03 %	3.0 %	5.0 %
HCT [%]	0.32 %	4.9 %	8.3 %	−0.19 %	2.9 %	5.3 %
MCV [fl]	−0.18 %	1.9 %	2.8 %	0.19 %	2.0 %	3.8 %
MCH [pg]	−0.02 %	3.2 %	5.1 %	0.71 %	6.5 %	5.1 %
MCHC [g/dl]	−0.20 %	2.8 %	3.4 %	0.42 %	6.0 %	3.1 %
PLT [10³/µl]	2.91 %	21.2 %	37.5 %	0.53 %	17.3 %	32.0 %
LYM [10³/µl]	−3.12 %	30.2 %	47.9 %	−4.90 %	30.6 %	48.8 %
Lymphocytes [%]	1.70 %	6.5 %	11.5 %	1.84 %	7.9 %	13.9 %
Neutrophils [%]	−8.65 %	28.1 %	48.2 %	−6.74 %	32.8 %	57.9 %
Monocytes [%]	0.95 %	38.7 %	56.1 %	2.61 %	32.3 %	59.0 %
Eosinophils [%]	−11.50 %	46.8 %	77.8 %	−3.07 %	45.2 %	78.7 %
Basophils [%]						
*Clinical biochemistry*						
ALP [µkat/l]	−4.16 %	19.5 %	30.1 %	−1.34 %	16.9 %	28.9 %
ALT [µkat/l]	−15.85 %	106.4 %	26.8 %	−1.47 %	28.5 %	44.7 %
AST [µkat/l]	−17.27 %	54.9 %	57.9 %	−29.40 %	57.6 %	86.9 %
ALB [g/l]	−1.97 %	9.2 %	14.5 %	−0.42 %	10.2 %	16.5 %
GLU [mmol/l]	−0.69 %	24.8 %	43.0 %	−3.24 %	18.9 %	30.1 %
CREA [µmol/l]	−0.88 %	15.3 %	26.6 %	−4.37 %	17.4 %	29.7 %
TP [g/l]	0.10 %	4.6 %	7.2 %	1.10 %	8.0 %	14.0 %
U [mmol/l]	−1.29 %	12.2 %	20.2 %	−1.39 %	12.8 %	20.5 %
CHOL [mmol/l]	−0.27 %	12.7 %	22.9 %	−0.40 %	14.2 %	23.8 %
Ca [mmol/l]	−1.09 %	7.8 %	15.0 %	−0.04 %	8.9 %	15.7 %
Cl [mmol/l]	−0.96 %	6.1 %	6.6 %	−0.04 %	4.0 %	6.8 %
K (mmol/l)	−1.11 %	11.8 %	18.5 %	−5.01 %	18.6 %	29.3 %
Na [mmol/l]	−1.15 %	6.7 %	8.0 %	−0.11 %	3.6 %	5.8 %
P [mmol/l]	−2.67 %	20.1 %	22.6 %	2.32 %	17.5 %	28.3 %
TRG [mmol/l]	−1.91 %	33.0 %	60.2 %	0.96 %	30.5 %	46.5 %
*Organ weights*						
Kidney (right) [%]	0.45 %	6.5 %	11.1 %	−0.50 %	5.9 %	10.5 %
Kidney (left) [%]	0.64 %	5.9 %	10.4 %	−0.30 %	6.4 %	11.6 %
Spleen [%]	−0.61 %	9.1 %	16.7 %	0.04 %	11.5 %	20.4 %
Liver [%]	−1.53 %	7.7 %	12.3 %	0.20 %	7.8 %	12.3 %
Adrenal gland (right) [%]	1.05 %	16.3 %	29.2 %	0.57 %	14.8 %	26.9 %
Adrenal gland (left) [%]	−2.66 %	14.4 %	16.5 %	−0.44 %	11.2 %	20.1 %
Lung [%]	−1.24 %	10.0 %	15.3 %	0.07 %	8.1 %	12.8 %
Heart [%]	−0.90 %	5.3 %	9.2 %	−0.95 %	6.8 %	12.1 %
Thymus [%]	−2.59 %	18.9 %	31.7 %	−1.64 %	13.5 %	21.5 %
Pancreas [%]	1.55 %	14.7 %	24.3 %	−1.46 %	15.5 %	23.7 %
Uterus [%]				0.38 %	17.5 %	28.9 %
Ovary (right) [%]				2.74 %	18.6 %	29.2 %
Ovary (left) [%]				2.85 %	19.2 %	31.9 %
Testis (right) [%]	−0.55 %	7.8 %	14.7 %			
Testis (left) [%]	1.61 %	7.4 %	14.0 %			
Epididymis (right) [%]	0.09 %	7.9 %	14.8 %			
Epididymis (left) [%]	0.31 %	7.8 %	13.3 %			
Brain [%]	0.04 %	5.0 %	8.8 %	0.51 %	6.3 %	11.3 %

The corresponding natural variation of the data was described by two parameters of variability (coefficient of variation and the relative width of the 90 % central interpercentile range), which are listed for each parameter in Table 4, columns 3–4 and, 6–7.

The coefficient of variation in body weight at the beginning of the study was 4.3 % in males and 4.8 % in females, increasing over time up to 5.6 % in males and 7.3 % in females (week 13). The relative 90 % interpercentile range of the body weight measurements at the beginning of the study was 7.5 % of the mean in males and 8.2 % of the mean in females, also slightly increasing over time up to 9.1 % of the mean in males and 12.2 % of the mean in females (week 13).

In the case of the haematology parameters, the coefficient of variation ranged between 2 and 45 %, whereas the 90 % interpercentile interval varied between 3 and 78 % of the mean.

Clinical biochemistry parameters showed the highest variability: The coefficients of variation were about 5 to about 106 %, and the 90 % interpercentile intervals ranged from 4 to 86.7 % of the means. Relative organ weights had coefficients of variation of about 5–15 %, and 90 % interpercentile intervals ranged from 8 to 32 % of the means (Table 4).

### Assessment of the homogeneity of the historical control data set

In 94 % of the ANOVAs applied to all parameters (to body weight per week, to haematology and clinical biochemistry parameters as well as to relative organ weights of male and female rats), the hypothesis that the five study means were equal was rejected, i.e. for these parameters the mean in at least one study was different from the mean of at least one other study. The hypothesis of equal study means could not be rejected for MCV, eosinophils, ALT and epididymis (right) in males as well as for basophils and adrenal (right and left) in females.

**Fig. 3:**
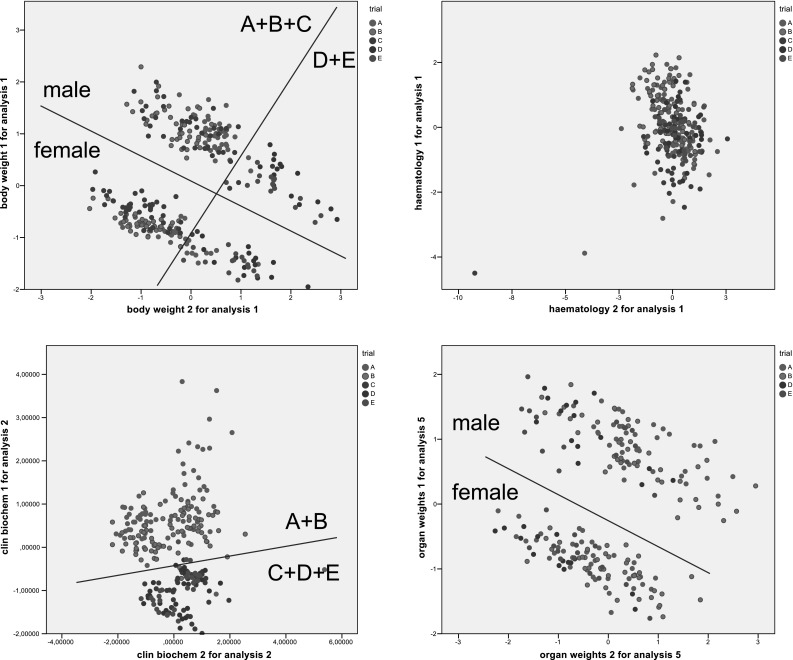
*Scatter plots* of principal components scores for body weight (*top left*), haematology parameters (*top right*), clinical biochemistry parameters (*bottom left*) and relative organ weights (*bottom right*)

PCA followed by CA for body and relative organ weights showed a clear separation of male and female rats into two clusters (Fig. 3, top left and bottom right). Additionally, in the case of the body weight, but not in the case of the relative organ weights, studies A, B and C were grouped in one cluster, separated from a second cluster including studies D and E. Furthermore, the clinical biochemistry parameters of studies A and B formed one cluster, separated from a second cluster including the parameters of studies C, D and E (Fig. 3, bottom left). Regarding the haematology parameters, there was no discrimination between the studies (Fig. 3, top right).

### Evaluation of the toxicological relevance of statistically significant differences

#### Consistency of statistically significant differences between GM and non-GM diet groups

##### Visual evaluation of the consistency of body weight data

**Fig. 4:**
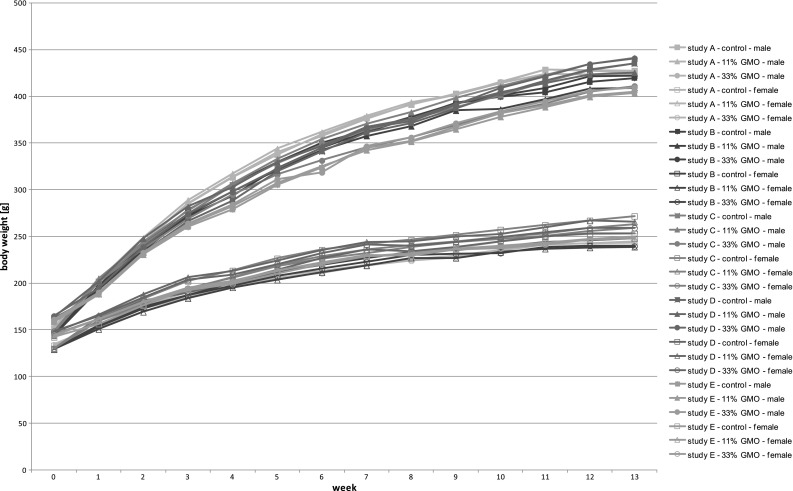
Line graphs of weight development in male and female animals from the control, 11 % GMO and 33 % GMO groups in the studies A–E

The weight development in each study is displayed in Fig. 4 (control, 11 % GMO and 33 % GMO groups). The curves run parallel to each other, thus indicating a comparable weight development in all studies. As already seen in the PCA graphs, all groups in studies D and E showed a slower body weight growth than in studies A, B and C.

##### Visual evaluation of the consistency of the other parameters

**Fig. 5:**
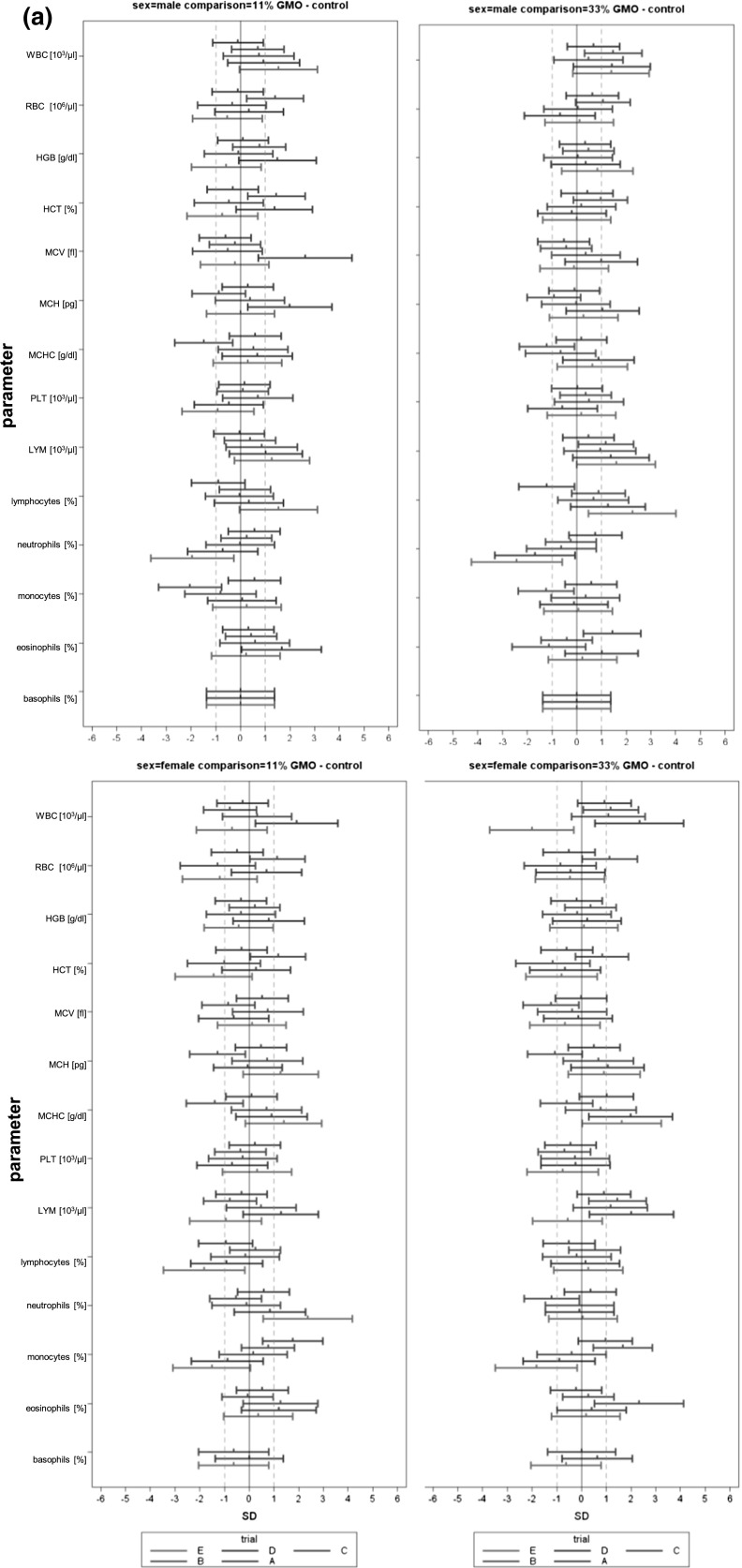

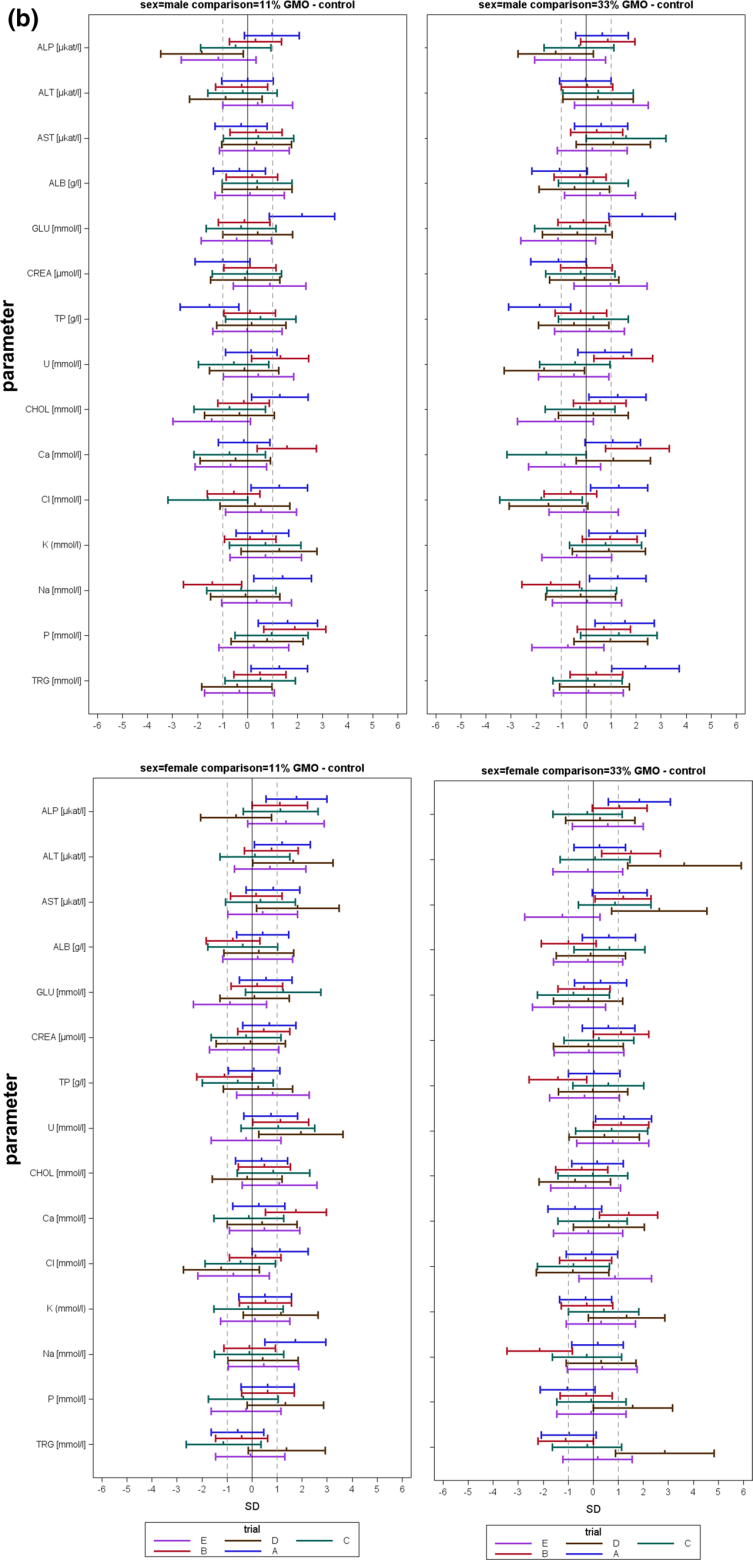

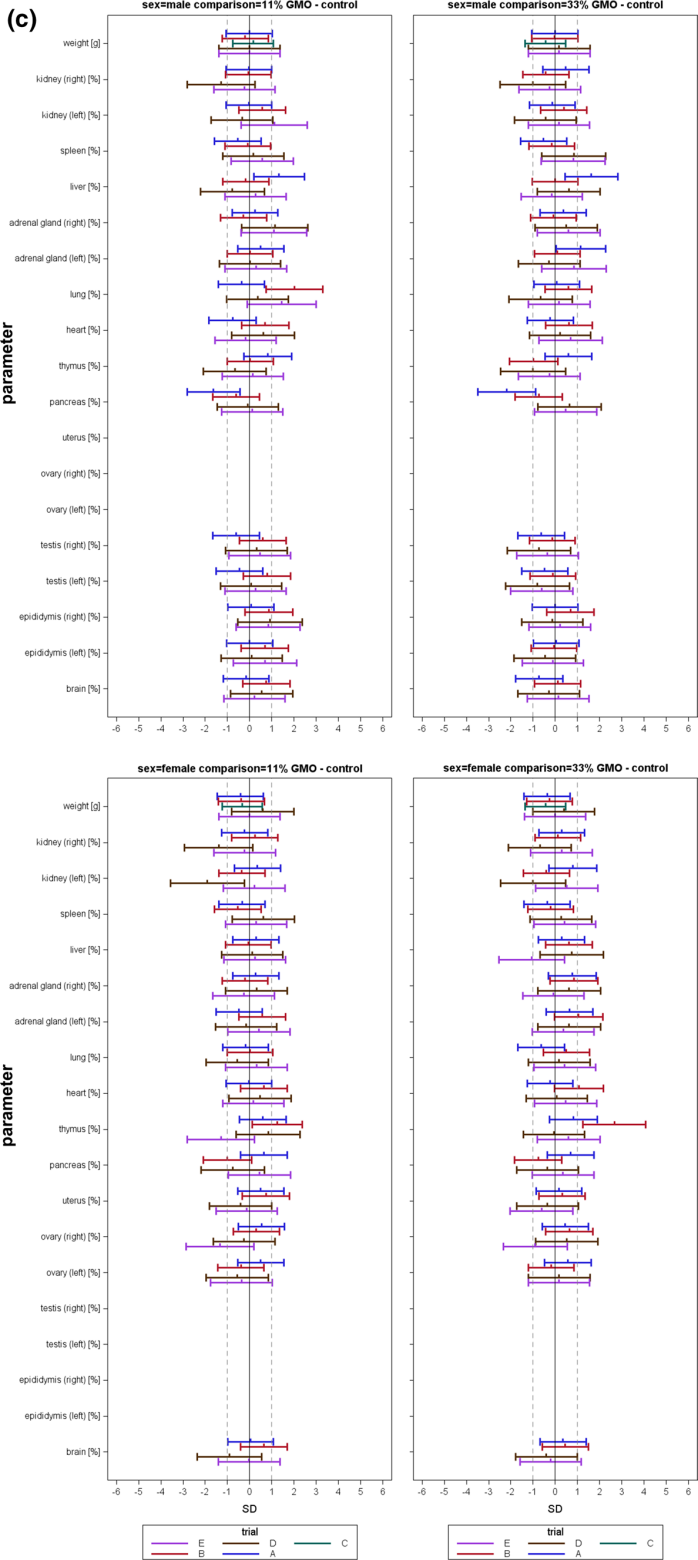
**a** Combined SES graphs for studies A-E, (left: 11 % GMO—control, right: 33 % GMO—control), haematology parameters in male (*top*) and female rats (*bottom*); **b** combined SES graphs for studies A-E, (left: 11 % GMO—control, right: 33 % GMO—control), clinical biochemistry parameters in male (*top*) and female rats (*bottom*); **c** combined SES graphs for studies A-E, (left: 11 % GMO—control, right: 33 % GMO—control), body weight and relative organ weights of male (*top*) and female rats (*bottom*)

The combined SES graphs of the five studies (both for 11% GMO–control and 33 % GMO–control comparisons) are shown in Fig. 5: 37.5 % of haematology, 25.0 % of clinical biochemistry parameters and 48.4 % of organ weights showed consistent difference values across the five studies. About 90 % of the SES confidence bars crossed the zero line (male comparison 11 % GMO–control: 89.0 %, male comparison 33 % GMO–control: 88.6 %, female comparison 11 % GMO–control: 92.3 %, female comparison 33 % GMO–control: 88.8 %), thereby indicating no statistically significant differences (see also Table 5). Single SES confidence interval bars moved to the right or to the left; such shifts were replicated once or twice for less than 1 % of haematology parameters, about 2 % of clinical chemistry parameters and less than 5 % of organ weights, but no confidence interval bar shift was reproduced in all five studies.

**Table 5:** Absolute and relative numbers of significant differences (based on SES estimates) in 11 % GMO−control and 33 % GMO−control group comparisons of the five feeding studies; total number of parameters: 45/44 (male/female; haematology and clinical biochemistry parameters as well as relative organ weights)

Comparison	Study				
GM maize variety from	Monsanto			Pioneer	
	A	C	D	B	E
*Absolute number of significant differences*					
11 % GMO–control					
Male	9	0	4	9	1
Female	4	0	5	7	2
33 % GMO–control					
Male	13	0	2	7	2
Female	2	1	6	12	2
*Relative number of significant differences*					
11 % GMO–control					
Male	20.0 %	0.0 %	8.9 %	20.0 %	2.2 %
Female	9.1 %	0.0 %	11.4 %	15.9 %	4.5 %
33 % GMO–control					
Male	28.9 %	0.0 %	4.4 %	15.6 %	4.4 %
Female	4.5 %	3.3 %	13.6 %	27.3 %	4.5 %

##### Evaluation of the consistency of the numbers of statistically significant differences

The total and relative numbers of statistically significant differences between the control and the test groups (based on SES estimates) in haematology, clinical biochemistry and organ weight measures (altogether 45 for female parameters and 44 for male parameters) are shown in Table 5. Up to 20 % statistically significant differences were observed in the studies A and B, about 10 % in study D and below 5 % in the studies C and E. Therefore, the number of observed statistically significant differences was not consistent across the studies.

##### Evaluation of the consistency of the difference values

The absolute 11 % GMO–control and 33 % GMO–control group differences for all parameters are shown in Table 6. Statistically significant negative differences are highlighted in italics, while statistically significant positive differences are highlighted in bold. Differences were not replicated across the studies (i.e. the columns), and in the case of some parameters even opposite differences (italics/bold) were observed. The absolute values of statistically significant differences often deviated extremely from the differences in the other studies. Taken together, there was no single difference between a control and a GMO group that was consistent across all five studies.

**Table 6:** Consistency of the difference values and significances (based on SES confidence intervals) across studies (statistically significant differences are marked in bold: GMO group mean > control group mean, or in italics: GMO group mean < control group mean)

Parameter	11 % GMO–control					33 % GMO–control				
	Monsanto			Pioneer		Monsanto			Pioneer	
	Study *A*	Study *C*	Study *D*	Study *B*	Study *E*	Study *A*	Study *C*	Study *D*	Study *B*	Study *E*
A: Male rats										
*Haematology*										
WBC [10^3^/µl]	−0.231	1.080	2.390	1.125	2.550	1.706	0.550	2.340	**2.675**	1.270
RBC [10^6^/µl]	−0.047	−0.136	0.056	**0.368**	−0.140	0.260	0.018	−0.185	0.329	0.022
HGB [g/dl]	0.138	−0.040	0.640	0.294	−0.300	0.406	0.030	0.160	0.213	0.170
HCT [%]	−0.750	−1.030	1.360	**1.831**	−1.030	0.869	0.350	−0.190	1.550	0.000
MCV [fl]	−0.663	−0.410	**1.290**	−0.225	−0.300	−0.750	0.360	1.080	−0.331	−0.110
MCH [pg]	0.288	0.310	**0.660**	−0.456	0.000	−0.100	−0.010	0.660	−0.463	0.180
MCHC [g/dl]	0.913	0.850	0.350	*−0.688*	0.160	0.288	−0.300	0.530	−*0.650*	0.380
PLT [10^3^/µl]	30.375	88.900	−73.100	5.938	−56.100	4.188	70.600	−46.400	24.063	9.800
LYM [10^3^/µl]	−0.100	0.940	1.840	0.525	1.160	0.938	1.020	1.650	**1.713**	0.830
Lymphocytes [%]	−1.844	−0.200	1.200	0.594	5.800	−*4.031*	2.700	4.400	2.063	**5.000**
Neutrophils [%]	1.094	−0.050	−2.800	0.719	−*6.250*	2.188	−2.200	−*5.100*	−0.438	−*5.300*
Monocytes [%]	0.594	−0.500	0.050	−*1.688*	0.200	0.625	0.300	−0.100	−*1.313*	0.050
Eosinophils [%]	0.188	0.750	**1.550**	0.375	0.250	**1.219**	−0.800	0.800	−0.281	0.250
Basophils [%]	0.000	0.000	0.000	0.000	0.000	0.000	0.000	0.000	0.000	0.000
*Clinical biochemistry*										
ALP [µkat/l]	0.196	−0.084	−*0.424*	0.054	−0.238	0.128	−0.045	−0.266	0.162	−0.127
ALT [µkat/l]	0.000	−0.013	−0.030	−0.015	0.024	−0.001	0.043	0.028	0.002	0.035
AST [µkat/l]	−0.055	0.203	0.199	0.043	0.097	0.139	0.430	0.449	0.064	0.043
ALB [g/l]	−0.769	0.900	0.610	0.244	0.160	−2.350	0.720	−0.900	−0.338	0.640
GLU [mmol/l]	**2.333**	−0.197	0.217	−0.206	−0.314	**2.355**	−0.394	−0.173	−0.191	−0.877
CREA [µmol/l]	−3.213	−0.110	−0.710	0.488	3.140	−3.675	−1.220	−0.400	0.088	3.480
TP [g/l]	−*4.475*	1.420	0.340	0.244	−0.030	−*6.756*	0.720	−1.260	−0.663	0.170
U [mmol/l]	0.056	−0.330	−0.042	**0.583**	0.167	0.358	−0.241	*−0.542*	**0.831**	−0.176
CHOL [mmol/l]	**0.550**	−0.100	−0.077	−0.038	−0.325	**0.508**	−0.037	0.073	0.156	−0.249
Ca [mmol/l]	−0.034	−0.044	−0.021	**0.348**	−0.120	0.309	−0.058	0.042	**0.331**	−0.148
Cl [mmol/l]	**14.625**	−1.600	0.400	−2.563	1.000	**12.563**	−2.200	−1.600	−1.813	−0.100
K (mmol/l)	0.438	0.570	0.640	0.031	0.410	**0.800**	0.700	0.350	0.188	−0.150
Na [mmol/l]	**23.938**	−0.400	−0.200	−*5.411*	0.800	**18.563**	−0.300	−0.400	−*3.167*	0.100
P [mmol/l]	**0.568**	0.284	0.100	**0.333**	0.054	**0.484**	0.349	0.176	0.133	−0.156
TRG [mmol/l]	**0.264**	0.082	−0.065	0.188	−0.056	**0.336**	0.008	0.067	0.124	0.014
*Organ weights*										
Kidney (right) [%]	−0.001		−0.021	−0.001	−0.004	0.010		−0.015	−0.008	−0.003
Kidney (left) [%]	−0.001		−0.007	0.010	0.017	−0.002		−0.009	0.008	0.002
Spleen [%]	−0.011		0.002	−0.001	0.005	−0.010		0.013	−0.003	0.012
Liver [%]	**0.132**		−0.053	−0.038	0.023	**0.126**		0.063	−0.001	−0.015
Adrenal gland (right) [%]	0.000		0.001	0.000	0.001	0.000		0.000	0.000	0.000
Adrenal gland (left) [%]	0.000		0.000	0.000	0.000	**0.001**		0.000	0.000	0.000
Lung [%]	−0.013		0.010	**0.037**	0.022	0.002		−0.013	0.020	0.003
Heart [%]	−0.010		0.006	0.008	−0.003	−0.003		0.002	0.007	0.008
Thymus [%]	0.015		−0.005	0.001	0.002	0.013		−0.008	−0.015	−0.004
Pancreas [%]	−*0.026*		−0.002	−0.012	0.002	−*0.029*		0.013	−0.015	0.011
Testis (right) [%]	−0.024		0.008	0.029	0.017	−0.024		−0.022	−0.005	−0.005
Testis (left) [%]	−0.020		0.002	0.039	0.010	−0.021		−0.026	−0.004	−0.011
Epididymis (right) [%]	0.001		0.008	0.012	0.013	0.000		−0.002	0.013	0.001
Epididymis (left) [%]	0.000		0.001	0.011	0.009	0.000		−0.007	−0.001	−0.001
Brain [%]	−0.003		0.019	0.023	0.006	−0.018		−0.008	0.004	0.003
B: Female rats										
*Haematology*										
WBC [10^3^/µl]	−0.513	0.490	**4.240**	−1.194	−0.840	1.719	1.550	**4.210**	**1.738**	*−1.690*
RBC [10^6^/µl]	−0.174	−0.442	0.139	**0.343**	−0.326	−0.139	−0.302	−0.100	**0.312**	−0.152
HGB [g/dl]	−0.163	−0.320	0.290	0.069	−0.180	−0.081	−0.160	0.070	0.131	0.050
HCT [%]	−0.488	−2.030	0.310	**1.275**	−1.760	−0.775	−2.010	−0.720	0.869	−1.240
MCV [fl]	0.644	0.650	−0.620	−0.881	0.100	−0.019	−0.400	−0.160	*−1.181*	−0.510
MCH [pg]	0.269	2.220	−0.030	*−0.781*	0.610	0.250	2.060	0.370	−0.650	0.470
MCHC [g/dl]	0.044	3.660	0.390	*−0.819*	1.040	0.438	4.020	**0.750**	−0.400	1.150
PLT [10^3^/µl]	15.563	−42.900	−97.900	−29.125	30.100	−34.250	−43.700	−35.200	−78.313	−92.400
LYM [10^3^/µl]	−0.494	0.450	1.500	−0.894	−0.660	1.294	1.110	**2.150**	**1.700**	−0.490
Lymphocytes [%]	−2.563	−0.600	−5.000	0.656	*−8.600*	−1.906	−1.100	0.800	1.313	1.200
Neutrophils [%]	1.531	−0.400	4.550	−1.281	**9.800**	1.313	−0.400	−0.400	*−2.938*	0.200
Monocytes [%]	**0.656**	0.100	−0.600	0.625	−1.500	0.750	−0.300	−0.700	**1.500**	*−1.500*
Eosinophils [%]	0.375	0.950	1.050	−0.031	0.350	−0.156	**1.800**	0.250	0.125	0.150
Basophils [%]	0.000	−0.050	0.000	0.000	−0.050	0.000	0.000	0.050	0.000	−0.050
*Clinical biochemistry*										
ALP [µkat/l]	**0.268**	0.070	−0.080	0.085	0.191	**0.244**	−0.035	0.041	0.103	0.078
ALT [µkat/l]	**0.093**	0.035	**0.193**	0.036	0.108	0.011	0.019	**0.340**	**0.078**	−0.033
AST [µkat/l]	0.121	0.382	**1.532**	0.028	0.151	0.224	0.769	**1.865**	**0.184**	−0.431
ALB [g/l]	1.950	−0.910	0.740	−3.294	0.420	2.519	1.830	−0.360	−4.163	−0.450
GLU [mmol/l]	0.640	0.675	0.092	0.263	−0.435	0.379	−0.519	−0.130	−0.386	−0.533
CREA [µmol/l]	2.150	−1.330	−0.290	2.569	−1.450	2.663	1.560	−0.740	4.725	−0.940
TP [g/l]	0.400	−1.290	0.950	−7.450	1.400	0.150	1.690	−0.070	−*8.988*	−0.770
U [mmol/l]	0.288	0.432	**0.471**	**0.723**	−0.087	**0.546**	0.393	0.124	0.632	0.274
CHOL [mmol/l]	0.149	0.250	−0.050	0.172	0.198	0.062	−0.005	−0.216	−0.084	−0.068
Ca [mmol/l]	0.018	−0.006	0.019	**0.308**	0.022	−0.061	−0.001	0.030	**0.175**	−0.010
Cl [mmol/l]	4.563	−1.500	−1.700	0.313	−1.000	−0.250	−1.700	−1.100	−0.625	1.000
K (mmol/l)	0.138	−0.110	0.460	0.294	0.070	−0.106	0.350	1.120	−0.094	0.180
Na [mmol/l]	**6.938**	−0.300	0.600	−0.375	0.800	0.688	−0.600	0.500	−*4.750*	0.700
P [mmol/l]	0.151	−0.110	0.320	0.209	−0.086	−0.234	−0.028	0.546	−0.097	−0.028
TRG [mmol/l]	−0.074	−0.139	0.101	−0.085	−0.008	−0.137	−0.038	**0.211**	−0.160	0.009
*Organ weights*										
Kidney (right) [%]	−0.005		−0.029	0.005	−0.004	0.005		−0.016	0.003	0.005
Kidney (left) [%]	0.008		*−0.018*	−0.008	0.005	0.012		−0.009	−0.009	0.014
Spleen [%]	−0.006		0.017	−0.011	0.005	−0.006		0.004	−0.006	0.009
Liver [%]	0.030		0.014	−0.006	0.022	0.028		0.086	0.083	−0.130
Adrenal gland (right) [%]	0.001		0.001	0.000	−0.001	0.001		0.002	0.002	0.000
Adrenal gland (left) [%]	−0.001		0.000	0.001	0.001	0.001		0.001	0.001	0.001
Lung [%]	−0.007		−0.014	0.000	0.008	−0.019		0.004	0.029	0.012
Heart [%]	0.000		0.007	0.007	0.003	−0.005		0.001	0.015	0.008
Thymus [%]	0.011		0.018	**0.020**	−0.016	0.016		−0.001	**0.028**	0.007
Pancreas [%]	0.017		−0.029	−0.024	0.008	0.017		−0.015	−0.016	0.014
Uterus [%]	0.015		−0.012	0.030	−0.004	0.004		−0.012	0.019	−0.017
Ovary (right) [%]	0.003		−0.001	0.001	−0.002	0.002		0.001	0.003	−0.002
Ovary (left) [%]	0.002		−0.001	−0.001	−0.001	0.003		0.000	−0.001	0.000
Brain [%]	0.003		−0.042	0.021	−0.002	0.022		−0.020	0.015	−0.012

Body weight values are not included, since the body weight development (not the week−by−week body weight) was analysed

#### Comparison of the values observed in GM maize-fed groups with the historical control data baseline

The means and SD of the historical control data, as shown in Table 3, are included in columns 2–3 of Table 7, and the derived simplified equivalence ranges (mean + SD, mean − SD) are listed in columns 4–5. Additionally, the means of the GM group parameters of all five studies are shown, and mean values laying within the simplified equivalence limits are highlighted in italics .

**Table 7:** Simplified equivalence limits (mean ± SD) for historical control data and GM group means’ fit−ins (italics: GM group mean falls within the simplified historical control equivalence range)

Parameter	Mean	SD	Equivalence limits		11 % GMO groups					33 % GMO groups				
			Lower	Upper	Study *A*	Study *B*	Study *C*	Study *D*	Study *E*	Study *A*	Study *B*	Study *C*	Study *D*	Study *E*
A: Male rats														
*Body weight*														
Week 0 [g]	149.9	6.5	143.4	156.4	*152.6*	*144.8*	*147.9*	163.5	159.3	*151.9*	*143.5*	*147.4*	164.8	161.1
Week 1 [g]	198.1	7.8	190.3	205.9	*203.2*	*194.3*	*205.3*	*202.0*	188.6	*202.5*	*192.9*	*201.1*	*194.3*	190.1
Week 2 [g]	240.1	9.1	231.0	249.2	*248.3*	*237.5*	*243.9*	*247.8*	230.6	*244.2*	*235.0*	*236.9*	*238.8*	*232.6*
Week 3 [g]	276.4	11.9	264.5	288.3	289.1	*273.0*	*277.9*	*282.0*	262.1	*285.8*	*271.8*	*268.9*	*274.0*	264.0
Week 4 [g]	303.5	14.9	288.6	318.4	*317.5*	*297.8*	*306.8*	*302.4*	282.4	*313.8*	*293.7*	*294.6*	*297.8*	284.2
Week 5 [g]	328.4	15.4	313.0	343.8	344.2	*321.5*	*332.9*	*329.5*	307.4	*340.1*	*322.8*	*316.4*	*322.7*	311.4
Week 6 [g]	348.4	17.1	331.3	365.5	*361.8*	*342.3*	*353.9*	*347.0*	325.4	*357.5*	*345.3*	*331.4*	*344.6*	318.7
Week 7 [g]	364.6	18.4	346.2	383.0	*379.3*	*357.8*	*370.7*	*367.1*	342.5	*375.9*	*362.4*	345.3	*366.0*	*346.6*
Week 8 [g]	377.6	20.4	357.2	398.0	*394.0*	*368.1*	*383.3*	*376.2*	351.6	*391.7*	*376.5*	356.1	*373.4*	356.0
Week 9 [g]	391.1	20.7	370.4	411.8	*401.5*	*384.9*	*398.5*	*391.9*	364.9	*401.7*	*393.4*	369.2	*391.1*	*371.4*
Week 10 [g]	401.3	21.2	380.1	422.5	*414.3*	*386.4*	*410.8*	*404.2*	378.2	*415.7*	*401.2*	*381.8*	*408.9*	*383.7*
Week 11 [g]	411.9	22.7	389.2	434.6	*424.1*	*397.0*	*423.1*	*413.9*	388.6	*428.9*	*409.0*	*392.6*	*421.2*	*394.8*
Week 12 [g]	419.7	22.4	397.3	442.1	*423.8*	*408.1*	*434.9*	*423.5*	*399.6*	*427.5*	*421.2*	*405.2*	*434.7*	*405.8*
Week 13 [g]	422.9	23.5	399.4	446.4	*423.5*	*409.2*	*441.5*	*425.6*	*403.4*	*425.6*	*422.4*	*411.2*	*440.9*	*409.8*
*Haematology*														
WBC [10^3^/µl]	10.14	2.88	7.26	13.02	*11.41*	*10.57*	*8.24*	*11.37*	*10.67*	13.35	*12.12*	*7.71*	*11.32*	*9.39*
RBC [10^6^/µl]	8.30	0.40	7.90	8.70	*8.33*	8.86	*7.98*	*8.27*	*8.06*	*8.64*	8.82	*8.14*	*8.03*	*8.22*
HGB [g/dl]	16.09	0.83	15.26	16.92	*16.06*	*16.69*	*16.05*	*16.49*	*15.86*	*16.33*	*16.61*	*16.12*	*16.01*	*16.33*
HCT [%]	45.98	2.25	43.73	48.23	*45.86*	49.04	*43.87*	*45.74*	*44.26*	*47.48*	48.76	*45.25*	*44.19*	*45.29*
MCV [fl]	55.40	1.04	54.36	56.44	*55.08*	*55.38*	*54.91*	*55.34*	*54.96*	*54.99*	*55.27*	*55.68*	*55.13*	*55.15*
MCH [pg]	19.40	0.62	18.78	20.02	*19.30*	*18.85*	20.17	*19.97*	*19.71*	*18.91*	*18.84*	*19.85*	*19.97*	*19.89*
MCHC [g/dl]	35.02	0.97	34.05	35.99	*35.03*	34.04	36.79	36.06	*35.85*	*34.40*	*34.08*	*35.64*	36.24	36.07
PLT [10^3^/µl]	751.64	159.51	592.13	911.15	*686.19*	*844.06*	*766.90*	*710.00*	*706.40*	*660.00*	*862.19*	*748.60*	*736.70*	*772.30*
LYM [10^3^/µl]	8.05	2.43	5.62	10.48	*9.33*	*8.69*	*5.87*	*8.50*	*7.15*	*10.36*	*9.88*	*5.95*	*8.31*	*6.82*
Lymphocytes [%]	77.31	5.05	72.26	82.36	*79.28*	*79.25*	70.80	*76.00*	*78.30*	*77.09*	*80.72*	*73.70*	*79.20*	*77.50*
Neutrophils [%]	17.51	4.91	12.60	22.42	*15.13*	*15.63*	23.85	*18.00*	*17.55*	*16.22*	*14.47*	*21.70*	*15.70*	*18.50*
Monocytes [%]	3.47	1.34	2.13	4.81	*4.06*	*3.13*	*2.25*	*2.95*	2.10	*4.09*	*3.50*	*3.05*	*2.80*	1.95
Eosinophils [%]	1.69	0.79	0.90	2.48	*1.53*	*1.97*	3.10	3.05	*2.05*	2.56	*1.31*	*1.55*	*2.30*	*2.05*
Basophils [%]	0.00	0.00	0.00	0.00			*0.00*	*0.00*	*0.00*			*0.00*	*0.00*	*0.00*
*Clinical biochemistry*														
ALP [µkat/l]	1.35	0.26	1.09	1.61	*1.49*	*1.39*	*1.10*	1.09	*1.25*	*1.42*	*1.50*	*1.13*	*1.24*	*1.36*
ALT [µkat/l]	0.63	0.67	−0.04	1.30	*0.53*	*0.60*	*0.46*	*0.46*	*0.49*	*0.53*	*0.61*	*0.52*	*0.52*	*0.50*
AST [µkat/l]	1.44	0.79	0.65	2.23	*1.17*	*1.00*	2.27	2.56	2.33	*1.36*	*1.02*	2.50	2.81	2.28
ALB [g/l]	34.35	3.17	31.18	37.52	*31.72*	*33.85*	39.34	37.94	*37.34*	30.14	*33.27*	39.16	37.63	38.78
GLU [mmol/l]	8.63	2.14	6.49	10.77	10.80	*9.21*	6.26	5.72	5.87	10.83	*9.22*	6.06	5.33	5.30
CREA [µmol/l]	43.31	6.61	36.70	49.92	*37.28*	*41.68*	*48.10*	*43.91*	*45.91*	*36.82*	*41.28*	*46.99*	*44.22*	*46.25*
TP [g/l]	60.96	2.80	58.16	63.76	57.02	*59.83*	64.95	*61.56*	*61.93*	54.74	*58.93*	64.25	*62.20*	*63.30*
U [mmol/l]	6.11	0.75	5.36	6.86	*6.14*	*6.20*	*5.88*	5.30	*5.52*	*6.44*	*6.45*	*5.97*	4.80	5.18
CHOL [mmol/l]	2.29	0.29	2.00	2.58	2.74	*2.26*	1.93	*2.07*	1.90	2.69	*2.45*	1.99	*2.22*	1.97
Ca [mmol/l]	2.57	0.20	2.37	2.77	*2.67*	*2.75*	*2.45*	*2.45*	*2.48*	3.01	*2.73*	*2.44*	*2.51*	*2.45*
Cl [mmol/l]	108.03	6.64	101.39	114.67	124.13	*107.25*	*103.20*	*103.20*	*103.40*	122.06	*108.00*	*102.60*	101.20	*102.30*
K (mmol/l)	4.83	0.57	4.26	5.40	*5.21*	*4.50*	*5.37*	*4.82*	*4.92*	5.58	*4.66*	5.50	5.44	*4.93*
Na [mmol/l]	148.97	9.94	139.03	158.91	174.31	*144.53*	*144.10*	*142.30*	*142.20*	168.94	*146.77*	*144.20*	*142.10*	*141.50*
P [mmol/l]	2.60	0.52	2.08	3.12	3.23	*2.84*	*2.36*	*2.48*	*2.48*	3.15	*2.64*	*2.43*	*2.56*	*2.27*
TRG [mmol/l]	0.72	0.24	0.48	0.96	*0.75*	*0.84*	*0.88*	*0.74*	*0.77*	*0.82*	*0.78*	*0.80*	*0.87*	*0.84*
*Organ weights*														
Kidney (right) [%]	0.283	0.018	0.265	0.301	*0.274*	*0.292*		0.258	*0.281*	*0.285*	*0.285*		0.265	*0.281*
Kidney (left) [%]	0.283	0.017	0.266	0.300	*0.287*	*0.293*		0.266	*0.287*	*0.286*	*0.291*		0.264	*0.272*
Spleen [%]	0.185	0.017	0.168	0.202	*0.176*	*0.196*		*0.170*	*0.176*	*0.178*	*0.194*		*0.181*	*0.183*
Liver [%]	2.214	0.169	2.045	2.383	*2.258*	*2.267*		*2.068*	*2.154*	*2.253*	*2.304*		*2.184*	*2.116*
Adrenal gland (right) [%]	0.006	0.001	0.005	0.007	*0.007*	*0.006*		*0.006*	0.007	*0.007*	*0.006*		*0.005*	*0.007*
Adrenal gland (left) [%]	0.007	0.001	0.006	0.008	*0.007*	*0.007*		*0.007*	*0.007*	*0.007*	*0.007*		*0.007*	*0.007*
Lung [%]	0.330	0.033	0.297	0.363	*0.344*	*0.340*		*0.317*	*0.321*	*0.359*	*0.324*		0.294	*0.302*
Heart [%]	0.230	0.012	0.218	0.242	*0.232*	*0.233*		*0.234*	*0.230*	*0.239*	*0.232*		*0.229*	*0.241*
Thymus [%]	0.100	0.019	0.081	0.119	*0.111*	0.121		*0.087*	*0.097*	*0.109*	*0.105*		*0.085*	*0.091*
Pancreas [%]	0.134	0.020	0.114	0.154	*0.115*	*0.129*		*0.134*	*0.142*	0.112	*0.126*		*0.149*	*0.151*
Testis (right) [%]	0.468	0.037	0.431	0.505	*0.452*	*0.501*		*0.442*	*0.472*	*0.452*	*0.468*		0.412	*0.450*
Testis (left) [%]	0.468	0.035	0.433	0.503	*0.454*	0.514		*0.437*	*0.472*	*0.453*	*0.471*		0.409	*0.451*
Epididymis (right) [%]	0.152	0.012	0.140	0.164	*0.157*	*0.159*		*0.152*	0.166	*0.156*	*0.160*		*0.142*	*0.154*
Epididymis (left) [%]	0.154	0.012	0.142	0.166	*0.159*	*0.162*		*0.147*	*0.161*	*0.159*	*0.150*		0.139	*0.151*
Brain [%]	0.529	0.027	0.502	0.556	*0.524*	*0.545*		*0.533*	*0.552*	*0.509*	*0.526*		*0.506*	*0.550*
B: Female rats														
*Body weight*														
Week 0 [g]	132.4	6.4	126.0	138.8	*130.4*	*129.0*	*130.0*	146.7	143.6	*134.1*	*129.8*	*130.0*	148.6	144.5
Week 1 [g]	156.9	7.1	149.8	164.0	*152.5*	*150.6*	*160.0*	165.8	*161.6*	*154.4*	*153.8*	*159.5*	164.9	*161.3*
Week 2 [g]	176.7	8.7	168.0	185.4	*172.9*	*169.0*	*177.1*	187.9	*180.0*	*173.4*	*172.5*	*176.7*	*184.3*	*179.0*
Week 3 [g]	192.1	10.8	181.3	202.9	*185.3*	*183.6*	*194.0*	206.4	*194.8*	*184.8*	*186.9*	*194.6*	203.5	*192.6*
Week 4 [g]	203.1	11.7	191.4	214.8	*198.0*	*195.3*	*206.2*	*212.8*	*202.4*	*196.6*	*197.1*	*205.5*	*208.8*	*199.1*
Week 5 [g]	214.5	12.2	202.3	226.7	*208.0*	*203.5*	*219.3*	*224.1*	*213.8*	*205.8*	*207.4*	*218.0*	*220.3*	*210.5*
Week 6 [g]	222.8	13.2	209.6	236.0	*213.1*	*211.5*	*228.9*	*235.1*	*222.3*	*210.7*	*215.5*	*226.1*	*231.9*	*220.0*
Week 7 [g]	229.7	14.0	215.7	243.7	*219.4*	*218.7*	*235.7*	244.3	*229.9*	*218.6*	*222.7*	*232.4*	*241.1*	*228.9*
Week 8 [g]	235.4	14.2	221.2	249.6	*225.7*	*227.0*	*240.4*	*244.3*	*229.2*	*224.1*	*229.9*	*238.6*	*240.2*	*229.9*
Week 9 [g]	239.0	14.9	224.1	253.9	*229.2*	*226.9*	*244.9*	*250.1*	*235.9*	*227.7*	*231.1*	*244.0*	*243.9*	*235.9*
Week 10 [g]	244.1	15.8	228.3	259.9	*235.2*	*233.1*	*249.4*	*252.9*	*237.9*	*234.8*	*232.3*	*247.5*	*249.1*	*237.2*
Week 11 [g]	249.4	16.4	233.0	265.8	*241.3*	*236.7*	*253.4*	*260.1*	*242.6*	*241.2*	*238.7*	*250.6*	*254.8*	*241.4*
Week 12 [g]	251.7	17.6	234.1	269.3	*242.5*	*238.0*	*259.3*	*266.8*	*246.6*	*242.8*	*239.9*	*256.0*	*259.2*	*246.7*
Week 13 [g]	253.4	18.4	235.0	271.8	*244.5*	*238.8*	*262.9*	*265.6*	*248.1*	*243.1*	*239.7*	*259.2*	*259.2*	*247.8*
*Haematology*														
WBC [10^3^/µl]	7.84	2.07	5.77	9.91	*8.34*	*7.59*	5.65	10.76	*8.27*	10.57	10.52	*6.71*	10.73	*7.42*
RBC [10^6^/µl]	7.67	0.29	7.38	7.96	*7.48*	*7.95*	7.27	*7.73*	*7.54*	*7.52*	*7.92*	*7.41*	*7.49*	*7.71*
HGB [g/dl]	15.45	0.47	14.98	15.92	*15.23*	*15.62*	14.83	*15.81*	*15.51*	*15.31*	*15.68*	*14.99*	*15.59*	*15.74*
HCT [%]	43.83	1.28	42.55	45.11	*43.20*	45.34	41.69	*43.78*	*42.76*	*42.91*	*44.93*	41.71	*42.75*	*43.28*
MCV [fl]	57.19	1.15	56.04	58.34	*57.75*	*57.05*	*57.39*	*56.65*	*56.75*	*57.09*	*56.75*	*56.34*	*57.11*	*56.14*
MCH [pg]	20.06	1.31	18.75	21.37	*20.38*	*19.68*	*20.42*	*20.44*	*20.58*	*20.36*	*19.81*	*20.26*	*20.84*	*20.44*
MCHC [g/dl]	35.05	2.09	32.96	37.14	*35.29*	*34.48*	*35.59*	*36.12*	*36.30*	*35.68*	*34.89*	*35.95*	*36.48*	*36.41*
PLT [10^3^/µl]	783.61	135.85	647.76	919.46	*860.38*	*812.31*	*656.50*	610.10	*770.50*	*810.56*	*763.13*	*655.70*	*672.80*	*648.00*
LYM [10^3^/µl]	5.99	1.84	4.15	7.83	*6.76*	*5.99*	3.76	*5.78*	*4.84*	8.55	8.59	*4.42*	*6.43*	*5.01*
Lymphocytes [%]	78.07	6.15	71.92	84.22	*80.31*	*82.91*	69.85	64.50	65.10	*80.97*	*83.56*	69.35	70.30	*74.90*
Neutrophils [%]	17.56	5.75	11.81	23.31	*14.97*	*13.03*	24.60	31.45	31.30	*14.75*	11.38	24.60	26.50	*21.70*
Monocytes [%]	2.68	0.86	1.82	3.54	*2.84*	*2.66*	*2.75*	*1.90*	1.70	*2.94*	*3.53*	*2.35*	1.80	1.70
Eosinophils [%]	1.68	0.76	0.92	2.44	*1.88*	*1.38*	2.80	*2.15*	*1.90*	*1.34*	*1.53*	3.65	*1.35*	*1.70*
Basophils [%]	0.04	0.09	-0.05	0.13			*0.00*	*0.00*	*0.00*			*0.05*	*0.05*	*0.00*
*Clinical biochemistry*														
ALP [µkat/l]	0.59	0.10	0.49	0.69	0.86	*0.63*	*0.69*	*0.62*	*0.66*	0.83	*0.65*	*0.58*	0.74	*0.55*
ALT [µkat/l]	0.48	0.14	0.34	0.62	*0.54*	*0.54*	*0.52*	0.63	*0.61*	*0.46*	*0.58*	*0.51*	0.77	*0.47*
AST [µkat/l]	1.46	0.84	0.62	2.30	*1.09*	*0.96*	3.06	4.14	3.12	*1.19*	*1.12*	3.44	4.47	2.54
ALB [g/l]	42.75	4.38	38.37	47.13	*43.14*	37.69	*45.73*	47.19	47.18	*43.71*	36.83	48.47	*45.60*	*46.31*
GLU [mmol/l]	6.82	1.29	5.53	8.11	*8.05*	*6.87*	*7.29*	5.47	5.43	*7.79*	*6.22*	*6.10*	5.25	5.33
CREA [µmol/l]	42.67	7.40	35.27	50.07	*37.79*	*40.86*	*49.39*	*45.14*	*45.93*	*38.31*	*43.02*	52.28	*44.69*	*46.44*
TP [g/l]	68.62	5.47	63.15	74.09	*69.52*	62.79	*68.78*	74.12	76.34	*69.27*	61.26	*71.76*	*72.63*	74.17
U [mmol/l]	5.62	0.72	4.90	6.34	*5.46*	*5.92*	6.61	*5.49*	*5.43*	*5.72*	*5.83*	6.57	*5.14*	*5.80*
CHOL [mmol/l]	2.01	0.29	1.72	2.30	*2.29*	*2.07*	*2.13*	*1.99*	*1.99*	*2.20*	*1.81*	*1.87*	*1.83*	*1.72*
Ca [mmol/l]	2.59	0.23	2.36	2.82	2.84	*2.80*	*2.56*	*2.58*	*2.59*	*2.76*	*2.66*	*2.56*	*2.59*	*2.56*
Cl [mmol/l]	106.54	4.27	102.27	110.81	113.25	*108.56*	100.20	99.80	100.70	*108.44*	*107.63*	100.00	100.40	*102.70*
K (mmol/l)	4.42	0.82	3.60	5.24	*4.18*	*4.34*	*5.02*	*4.63*	*4.99*	*3.94*	*3.96*	5.48	*4.96*	*5.10*
Na [mmol/l]	148.16	5.37	142.79	153.53	156.19	*151.81*	141.60	*143.90*	142.30	*149.94*	*147.44*	141.30	*143.80*	142.20
P [mmol/l]	2.33	0.41	1.92	2.74	*2.64*	*2.52*	1.91	*2.03*	1.85	*2.26*	*2.22*	*1.99*	*2.25*	1.91
TRG [mmol/l]	0.52	0.16	0.36	0.68	*0.49*	*0.39*	*0.57*	0.74	0.70	*0.42*	0.31	*0.67*	0.85	0.71
*Organ weights*														
Kidney (right) [%]	0.316	0.019	0.297	0.335	*0.314*	*0.322*		0.288	0.293	*0.323*	*0.320*		*0.301*	*0.303*
Kidney (left) [%]	0.316	0.020	0.296	0.336	*0.327*	*0.314*		0.286	0.293	*0.332*	*0.313*		0.295	*0.303*
Spleen [%]	0.246	0.028	0.218	0.274	*0.243*	*0.249*		*0.230*	0.216	*0.243*	*0.254*		0.218	*0.220*
Liver [%]	2.508	0.196	2.312	2.704	*2.525*	*2.398*		*2.499*	*2.488*	*2.523*	*2.487*		*2.570*	*2.336*
Adrenal gland (right) [%]	0.014	0.002	0.012	0.016	*0.015*	*0.013*		*0.015*	*0.013*	*0.015*	*0.015*		*0.015*	*0.013*
Adrenal gland (left) [%]	0.015	0.002	0.013	0.017	*0.014*	*0.015*		*0.015*	*0.016*	*0.016*	*0.016*		*0.017*	*0.016*
Lung [%]	0.443	0.036	0.407	0.479	*0.466*	*0.431*		0.400	0.407	*0.453*	*0.459*		*0.418*	*0.410*
Heart [%]	0.281	0.019	0.262	0.300	*0.293*	*0.274*		*0.290*	0.277	*0.288*	*0.282*		*0.284*	*0.283*
Thymus [%]	0.131	0.018	0.113	0.149	*0.144*	0.152		*0.137*	*0.114*	*0.148*	0.160		*0.118*	*0.136*
Pancreas [%]	0.183	0.028	0.155	0.211	*0.198*	*0.164*		*0.166*	*0.182*	*0.199*	*0.172*		*0.180*	*0.188*
Uterus [%]	0.202	0.035	0.167	0.237	*0.223*	*0.236*		*0.195*	*0.184*	*0.213*	*0.225*		*0.195*	*0.170*
Ovary (right) [%]	0.025	0.005	0.020	0.030	*0.028*	*0.027*		0.018	0.017	*0.027*	*0.029*		0.020	0.017
Ovary (left) [%]	0.026	0.005	0.021	0.031	*0.028*	*0.025*		0.018	0.017	*0.028*	*0.026*		0.020	0.018
Brain [%]	0.835	0.052	0.783	0.887	*0.809*	*0.860*		*0.802*	*0.842*	*0.828*	*0.854*		*0.824*	*0.832*

In male rats, 81.5 % of the parameters measured in the 11 % GMO group and 81.8 % of the parameters measured in the 33 %GMO group were within the simplified equivalence interval, whereas in female rats 77.9 % of the parameters measured in the 11 % GMO group and 82.3 % of the parameters measured in the 33 % GMO group were within the simplified equivalence interval. No dose–response relationship could be observed.

Moreover, 11.3 % of all parameters measured in study A, 7.8 % of all parameters measured in study B, 25.0 % of all parameters measured in study C, 26.9 % of all parameters measured in study D and 26.0 % of all parameters measured in study E were outside the equivalence limits.

### Assessment of the statistical power of the studies

Table 8, part a, specifies the achievable statistical power to detect effect sizes of one SD (corresponds to √2 SD with 2 animals/cage) with the given sample sizes proposed by several internationally recognized test guidelines. Table 8, part b, specifies the relative effect sizes (in SD units) detectable with a power of 0.9 for the same given sample sizes.

**Table 8:** (a) Achievable power to detect effect sizes of one SD (corresponds to√2 SD with 2 animals/cage) with the given sample sizes proposed by commonly applied test guidelines/recommendations and (b) required biologically relevant effect size in SD units (sensitivity) detectable with 2 animals/cage (test probability *α* = 0.05 for both tables)

	OECD Test Guideline on repeated−dose 90−day oral toxicity studies	EFSA guidance on repeated−dose 90−day oral toxicity studies with whole food/feed	OECD Test guideline on chronic toxicity studies
(a)			
Number of animals per group and sex	10	16	20
Number of cages per group and sex	5	8	10
Power to detect effect sizes of one SD (corresponds to √2 SD with 2 animals/cage)	0.50	0.75	0.85
(b)			
Number of animals per group and sex	10	16	20
Number of cages per group and sex	5	8	10
Detectable effect size in SD units (power of 0.9)	1.66	1.23	1.08
Detectable effect size in SD units (power of 0.8)	1.22	0.92	0.82

Combining the relative effect sizes of Table 8, part b, and the historical control data, absolute and from a practical point of view relevant effects sizes were calculated. The values for given sample sizes of (i) 10 animals per group and sex (OECD 2009b), (ii) 16 animals per group and sex (EFSA 2011 b) and (iii) 20 animals per group and sex (OECD 1998) in the case of two animals per cage are listed in Table 9. For the testing facility with the historical data background, these values represent the effect sizes that might be detected using statistical tools.

**Table 9:** Required effect sizes detectable by study designs with sample sizes of 5, 8 or 10 cages á 2 animals per group and sex, power = 0.8 and *α* = 0.05, based on historical control data

Parameter	Male				Female			
	One SD	*n* = 5×2	*n* = 8×2	*n* = 10 × 2	One SD	*n* = 5×2	*n* = 8 × 2	*n* = 10 × 2
*Body weight*								
Week 0 [g]	6.5	7.9	6.0	5.3	6.4	7.8	5.9	5.2
Week 1 [g]	7.8	9.5	7.2	6.4	7.1	8.7	6.5	5.8
Week 2 [g]	9.1	11.1	8.4	7.5	8.7	10.6	8.0	7.1
Week 3 [g]	11.9	14.5	10.9	9.8	10.8	13.2	9.9	8.9
Week 4 [g]	14.9	18.2	13.7	12.2	11.7	14.3	10.8	9.6
Week 5 [g]	15.4	18.8	14.2	12.6	12.2	14.9	11.2	10.0
Week 6 [g]	17.1	20.9	15.7	14.0	13.2	16.1	12.1	10.8
Week 7 [g]	18.4	22.4	16.9	15.1	14.0	17.1	12.9	11.5
Week 8 [g]	20.4	24.9	18.8	16.7	14.2	17.3	13.1	11.6
Week 9 [g]	20.7	25.3	19.0	17.0	14.9	18.2	13.7	12.2
Week 10 [g]	21.2	25.9	19.5	17.4	15.8	19.3	14.5	13.0
Week 11 [g]	22.7	27.7	20.9	18.6	16.4	20.0	15.1	13.4
Week 12 [g]	22.4	27.3	20.6	18.4	17.6	21.5	16.2	14.4
Week 13 [g]	23.5	28.7	21.6	19.3	18.4	22.4	16.9	15.1
*Hamematology*								
WBC [10^3^/nl]	2.88	3.51	2.65	2.36	2.07	2.53	1.90	1.70
RBC [10^6^4il]	0.40	0.49	0.37	0.33	0.29	0.35	0.27	0.24
HGB [g/dl]	0.83	1.01	0.76	0.68	0.47	0.57	0.43	0.39
HCT [%]	2.25	2.75	2.07	1.85	1.28	1.56	1.18	1.05
MCV [fl]	1.04	1.27	0.96	0.85	1.15	1.40	1.06	0.94
MCH [pg]	0.62	0.76	0.57	0.51	1.31	1.60	1.21	1.07
MCHC [g/dl]	0.97	1.18	0.89	0.80	2.09	2.55	1.92	1.71
PLT [10^3^/iil]	159.5	194.6	146.7	130.8	135.9	165.7	125.0	111.4
LYM [1 0^3^/iil]	2.43	2.96	2.24	1.99	1.84	2.24	1.69	1.51
Lymphocytes [%]	5.05	6.16	4.65	4.14	6.15	7.50	5.66	5.04
Neutrophils [%]	4.91	5.99	4.52	4.03	5.75	7.02	5.29	4.72
Monocytes [%]	1.34	1.63	1.23	1.10	0.86	1.05	0.79	0.71
Eosinophils [%]	0.79	0.96	0.73	0.65	0.76	0.93	0.70	0.62
Basophils [%]	0.00	0.00	0.00	0.00	0.09	0.11	0.08	0.07
*Clinical biochemistry*								
ALP [ukat/l]	0.26	0.32	0.24	0.21	0.10	0.12	0.09	0.08
ALT [ukat/l]	0.67	0.82	0.62	0.55	0.14	0.17	0.13	0.11
AST [ukat/l]	0.79	0.96	0.73	0.65	0.84	1.02	0.77	0.69
ALB [g/l]	3.17	3.87	2.92	2.60	4.38	5.34	4.03	3.59
GLU [mmol/l]	2.14	2.61	1.97	1.75	1.29	1.57	1.19	1.06
CREA [umol/l]	6.61	8.06	6.08	5.42	7.40	9.03	6.81	6.07
TP [g/l]	2.80	3.42	2.58	2.30	5.47	6.67	5.03	4.49
U [mmol/l]	0.75	0.92	0.69	0.62	0.72	0.88	0.66	0.59
CHOL [mmol/l]	0.29	0.35	0.27	0.24	0.29	0.35	0.27	0.24
Ca [mmol/l]	0.20	0.24	0.18	0.16	0.23	0.28	0.21	0.19
Cl [mmol/l]	6.64	8.10	6.11	5.44	4.27	5.21	3.93	3.50
K (mmol/l)	0.57	0.70	0.52	0.47	0.82	1.00	0.75	0.67
Na [mmol/l]	9.94	12.13	9.14	8.15	5.37	6.55	4.94	4.40
P [mmol/l]	0.52	0.63	0.48	0.43	0.41	0.50	0.38	0.34
TRG [mmol/l]	0.24	0.29	0.22	0.20	0.16	0.20	0.15	0.13
*Organ weight*								
Kidney (right) [%]	0.018	0.022	0.017	0.015	0.019	0.023	0.017	0.016
Kidney (left) [%]	0.017	0.021	0.016	0.014	0.020	0.024	0.018	0.016
Spleen [%]	0.017	0.021	0.016	0.014	0.028	0.034	0.026	0.023
Liver [%]	0.169	0.206	0.155	0.139	0.196	0.239	0.180	0.161
Adrenal gland (right) [%]	0.001	0.001	0.001	0.001	0.002	0.002	0.002	0.002
Adrenal gland (left) [%]	0.001	0.001	0.001	0.001	0.002	0.002	0.002	0.002
Lung [%]	0.033	0.040	0.030	0.027	0.036	0.044	0.033	0.030
Heart [%]	0.012	0.015	0.011	0.010	0.019	0.023	0.017	0.016
Thymus [%]	0.019	0.023	0.017	0.016	0.018	0.022	0.017	0.015
Pancreas [%]	0.020	0.024	0.018	0.016	0.028	0.034	0.026	0.023
Testis (right) [%]	0.037	0.045	0.034	0.030				
Testis (left) [%]	0.035	0.043	0.032	0.029				
Epididymis (right) [%]	0.012	0.015	0.011	0.010				
Epididymis (left) [%]	0.012	0.015	0.011	0.010				
Uterus [%]					0.035	0.043	0.032	0.029
Ovary (right) [%]					0.005	0.006	0.005	0.004
Ovary (left) [%]					0.005	0.006	0.005	0.004
Brain [%]	0.027	0.033	0.025	0.022	0.052	0.063	0.048	0.043

## Discussion

### 90-day feeding trials D and E

The feeding trials D and E were performed in parallel (i.e. all experimental conditions were strictly the same), while the studies A, B and C were performed at different points in time than the studies D and E in the same animal housing facility. Therefore, when discussing the relevance of statistically significant differences regarding a particular parameter between the control and the GMO-fed rats in the feeding studies D and E, it was considered pertinent to only compare the outcome of these two studies among themselves and not to include observations on alterations of the particular parameter in the feeding trials A, B and C.

There were no statistically significant differences between the mean body weights of the three experimental groups at any point in time of the feeding trials D and E in the case of male as well as female rats. Regarding the haematology parameters, three out of 14 parameters in male rats fed the 11 % GMO diet and one out of 14 parameters in male rats fed the 33 % GMO diet were significantly different from the control male animals in trial D, while one out of 14 parameters in female rats fed the 11 % GMO diet and three out of 14 parameters in female rats fed the 33 % GMO diet were significantly different from the control female animals in trial D. In the case of study E, one out of 14 parameters in male rats fed the 11 % GMO diet and two out of 14 parameters in male rats fed the 33 % GMO diet were significantly different from the control male animals, whereas two out of 14 parameters in female rats fed the 11 % GMO diet and the 33 % GMO diet were significantly different from the control female animals. It is important to note that the statistically significant changes in the haematology parameters in trial D were different from those observed in trial E with only one exception: The percentage of neutrophils decreased in male rats fed the 33 % GMO diet in both studies. In this context, the only haematological alterations that showed a tentative dose–effect relationship were the decrease in the percentage of neutrophils in male rats as well as the increase in the MCHC and LYM in female rats in trial D and the decrease in WBC in female rats in trial E, whereby the values in all four cases were within or close to the value range of the control rats. Thus, the described alterations in the haematology parameters are not considered to be relevant from a toxicological point of view. This conclusion is supported by the fact that after feeding the rats for 1 year with the diet containing 33 % MON810 maize no changes were observed in the four above-mentioned parameters in male and female rats (Zeljenková et al. 2016).

None of the clinical biochemistry parameters showed statistically significant differences in the three experimental groups of feeding trial E, while one out of 15 clinical biochemistry parameters in male rats and three out of 15 clinical biochemistry parameters in female rats were significantly different when the data from control and GMO-fed rats in the feeding trial D were compared. The increased ALT and AST activity in female rats were not accompanied by changes in the ALB and TP levels and/or by histopathological alterations in the liver and were not observed in female rats in the feeding trial E. An increased U level was observed in female rats fed the 11 % GMO diet but not the 33 % GMO diet, and the increased TRG level in the female rats fed the 33 % GMO diet was not accompanied by a lipid accumulation in any of the histologically examined organs and was not observed in trial E. Based on the above-mentioned observations, the described alterations in the clinical biochemistry parameters are not considered to be toxicologically relevant and are not related to the diets supplemented with the MON810 maize varieties.

A low number of histological changes, mostly inflammatory reactions and no pre-neoplastic/neoplastic lesions, were observed in the control and 33 % GMO groups in trials D and E, which is in accordance with the findings in the previously published 90-day feeding trials A and B (Zeljenková et al. 2014).

### Compilation of historical control data from groups fed 33 % non-GM maize-containing diets

In this study, historical control data regarding body weight development, haematology and clinical biochemistry parameters as well as relative organ weights of male and female Wistar Han RCC rats having been fed a diet containing 33 % non-GM maize at the animal housing facility of the Slovak Medical University (Bratislava, Slovakia) are presented. These data constitute the basis for future study designs, power analyses and study result assessments at this particular animal housing facility. The data refer to the six conventional varieties used in the five feeding studies (Table 2) and deliver useful information on the magnitude and variability of the measured parameters in male and female Wistar Han RCC rats being fed a diet containing 33 % non-GM maize for 90 days.

The ANOVA assessing the homogeneity of the historical control data showed a clear study effect. Moreover, in the case of body weight development and clinical biochemistry parameters, the data measured for diets containing the same maize variety are clearly clustered according to the studies (Fig. 3). This underlines the importance of comparing treatment groups (test and control groups) within studies. In the case of future feeding trials to be performed at the animal housing facility of the Slovak Medical University, the historical control data will help in determining whether the values of individual parameters measured in maize-fed rats show deviations from the corresponding normal range for rats held at the above-mentioned institution.

When analysing the historical control data, no statistically extreme values were excluded, in line with the approach in single study analyses where there were no technical reasons to do this. Consequently, the variability of some parameter values is high. In this context, the historical control data showed a reduced variability in studies C, D and E compared to studies A and B, which contained more extreme values.

### Evaluation of the toxicological relevance of statistically significant differences between control and test groups

#### Consistency of statistically significant differences

The methods to evaluate the consistency of statistically significant differences between control and test groups showed inconsistency both in the frequency of statistically significant differences and in the difference values. Statistically significant differences in one study were not reproduced in other studies, except in the following seven cases: (1) male, 33 % GMO—control, neutrophiles: study D and E; (2) female, 33 % GMO—control, WBC: study B and D; (3) female, 33 % GMO—control, LYM: study B and D; (4) female, 11 % GMO—control, ALT: study A and D; (5) female, 33 % GMO—control, ALT: study B and D; (6) female, 33 % GMO—control, AST: study B and D; (7) female, 11 % GMO—control, U: study B and D. Therefore, the great majority of the differences was only found in a single study, and the toxicological relevance of these statistically significant differences between control and test groups is questionable.

A higher percentage of statistically significant differences between rats fed the control diets and those fed the diets containing the MON810 maize was observed in the studies A and B than in the studies C, D and E. In this context, it has to be pointed out that the GRACE project partners did not find any evidence that this was due to major differences in the composition of the diets, to technical defects of the laboratory equipment used or to mistakes in the handling of the blood samples by the laboratory staff.

#### Comparison of the data sets from GM maize-fed rats with the historical control data

About 80 % of the individual parameter measurements in the GM maize-fed groups were within the simplified equivalence limits defined by the historical control data. If the parameter measurements fell outside the equivalence limits, the corresponding differences were statistically significant in 30 to 80 % of the cases.

The equivalence limits were calculated in a simplified way and had to be based on study-internal data; therefore, they only are rough estimates. A more refined equivalence testing procedure will be elaborated in the European Commission-funded project G-TwYST (GM Plants 2 Year Safety Testing, www.g-twyst.eu).

### Assessment of the statistical power of the studies

The post hoc power analysis revealed a power between 0.50 and 0.85 to detect an effect size of one SD in studies designed according to international guidelines (EFSA 2011b; OECD 1998, 2009) with samples sizes per group and sex of 10, 16 or 20, respectively (the experimental unit is the cage with two animals, i.e. 5, 8 or 10 cages á 2 animals). An effect size of one SD is not necessarily linked to a real toxicological relevant effect. The size of toxicological relevance in absolute or SD units should be considered separately for each parameter by toxicologists, largely based on previous experience. Therefore, based on the historical background data of the animal housing facility and its associated laboratories at the Slovak Medical University, the corresponding absolute effect sizes (in original units) were calculated (Table 9). They will constitute the basis for future study designs and power analyses. This is why they should be critically examined by toxicologists regarding their toxicological relevance.

## Electronic supplementary material

Below is the link to the electronic supplementary material.

Supplementary material 1 (DOCX 34 kb)

Supplementary material 2 (DOCX 34 kb)

Supplementary material 3 (DOCX 17 kb)
