# Supplementary material for: Variability of control data and relevance of observed group differences in five oral toxicity studies with genetically modified maize MON810 in rats
Source: Arch Toxicol. 2016 Oct 11;91(4):1977–2006. doi: 10.1007/s00204-016-1857-x (PMC5364247; doi:10.1007/s00204-016-1857-x)
Supplement: Supplementary file 1 — Supplementary material 1 (DOCX 34 kb) [file 204_2016_1857_MOESM1_ESM.docx]

**ESM-Table 1:** Trial D: mean ± standard deviations of body weight week-by-week, relative organ weights, haematology and clinical biochemistry parameters (significant groups differences of 11% GMO and 33% GMO to control are marked) as well as feed consumption and efficiency week-by-week

| **body weight** | **male** | | | **female** | | |
| --- | --- | --- | --- | --- | --- | --- |
|  | **control** | **11% GMO** | **33% GMO** | **control** | **11% GMO** | **33% GMO** |
| **Week 0 [g]** | 162.15 ± 3.43 | 163.46 ± 2.81 | 164.84 ± 2.85 | 144.69 ± 3.65 | 146.67 ± 4.81 | 148.56 ± 6.03 |
| **Week 1 [g]** | 188.18 ± 1.16 | 202.02 ± 2.91 | 194.25 ± 2.52 | 161.71 ± 4.92 | 165.80 ± 2.30 | 164.92 ± 6.10 |
| **Week 2 [g]** | 232.89 ± 3.87 | 247.75 ± 7.18 | 238.81 ± 3.95 | 180.99 ± 6.83 | 187.92 ± 6.83 | 184.31 ± 6.57 |
| **Week 3 [g]** | 266.28 ± 3.28 | 281.96 ± 13.33 | 274.04 ± 8.13 | 190.05 ± 19.95 | 206.37 ± 6.33 | 203.54 ± 8.96 |
| **Week 4 [g]** | 288.32 ± 4.92 | 302.39 ± 16.56 | 297.85 ± 13.30 | 201.96 ± 8.11 | 212.77 ± 5.79 | 208.82 ± 11.33 |
| **Week 5 [g]** | 320.00 ± 7.40 | 329.49 ± 20.76 | 322.75 ± 19.64 | 213.93 ± 8.41 | 224.07 ± 8.61 | 220.33 ± 12.92 |
| **Week 6 [g]** | 341.05 ± 9.36 | 346.96 ± 23.03 | 344.65 ± 22.30 | 227.05 ± 11.93 | 235.13 ± 11.29 | 231.88 ± 12.29 |
| **Week 7 [g]** | 361.36 ± 13.24 | 367.05 ± 24.80 | 366.05 ± 24.42 | 235.71 ± 13.23 | 244.26 ± 9.85 | 241.11 ± 15.55 |
| **Week 8 [g]** | 371.42 ± 13.12 | 376.18 ± 24.19 | 373.43 ± 29.08 | 233.88 ± 12.30 | 244.33 ± 10.20 | 240.16 ± 16.51 |
| **Week 9 [g]** | 387.46 ± 11.66 | 391.88 ± 24.91 | 391.13 ± 28.91 | 238.98 ± 14.59 | 250.05 ± 11.26 | 243.89 ± 17.21 |
| **Week 10 [g]** | 403.26 ± 14.00 | 404.24 ± 28.30 | 408.86 ± 27.02 | 244.47 ± 16.50 | 252.85 ± 10.67 | 249.08 ± 17.01 |
| **Week 11 [g]** | 417.14 ± 14.32 | 413.94 ± 31.75 | 421.17 ± 26.04 | 250.00 ± 17.64 | 260.09 ± 8.74 | 254.83 ± 18.17 |
| **Week 12 [g]** | 429.05 ± 14.16 | 424.46 ± 34.49 | 434.68 ± 26.37 | 253.02 ± 19.64 | 266.84 ± 8.72 | 259.16 ± 21.36 |
| **Week 13 [g]** | 435.24 ± 14.52 | 425.63 ± 32.34 | 440.93 ± 28.89 | 253.14 ± 17.80 | 265.63 ± 8.14 | 259.17 ± 17.89 |

| **organ weights** | **male** | | | | **female** | | | | |
| --- | --- | --- | --- | --- | --- | --- | --- | --- | --- |
|  | **control** | **11% GMO** | **33% GMO** | | **control** | **11% GMO** | | **33% GMO** | |
| **Kidney (right) [%]** | 0.28 ± 0.02 | 0.26 ± 0.01 | | 0.26 ± 0.00 | 0.32 ± 0.03 | | 0.29 ± 0.01 | | 0.30 ± 0.02 |
| **Kidney (left) [%]** | 0.27 ± 0.02 | 0.27 ± 0.02 | | 0.26 ± 0.02 | 0.30 ± 0.01 | | 0.29 ± 0.01^c^ | | 0.30 ± 0.01 |
| **Spleen [%]** | 0.17 ± 0.01 | 0.17 ± 0.02 | | 0.18 ± 0.02 | 0.21 ± 0.02 | | 0.23 ± 0.03 | | 0.22 ± 0.01 |
| **Liver [%]** | 2.12 ± 0.07 | 2.07 ± 0.07 | | 2.18 ± 0.13 | 2.48 ± 0.09 | | 2.50 ± 0.13 | | 2.57 ± 0.14 |
| **Adrenal (right) [%]** | 0.01 ± 0.00 | 0.01 ± 0.00 | | 0.01 ± 0.00 | 0.01 ± 0.00 | | 0.01 ± 0.00 | | 0.02 ± 0.00 |
| **Adrenal (left) [%]** | 0.01 ± 0.00 | 0.01 ± 0.00 | | 0.01 ± 0.00 | 0.02 ± 0.00 | | 0.02 ± 0.00 | | 0.02 ± 0.00 |
| **Lung [%]** | 0.31 ± 0.02 | 0.32 ± 0.03 | | 0.29 ± 0.01 | 0.41 ± 0.02 | | 0.40 ± 0.03 | | 0.42 ± 0.03 |
| **Heart [%]** | 0.23 ± 0.01 | 0.23 ± 0.01 | | 0.23 ± 0.01 | 0.28 ± 0.01 | | 0.29 ± 0.02 | | 0.28 ± 0.01 |
| **Thymus [%]** | 0.09 ± 0.01 | 0.09 ± 0.01 | | 0.08 ± 0.01 | 0.12 ± 0.02 | | 0.14 ± 0.02 | | 0.12 ± 0.01 |
| **Pancreas [%]** | 0.14 ± 0.01 | 0.13 ± 0.03 | | 0.15 ± 0.03 | 0.19 ± 0.05 | | 0.17 ± 0.01 | | 0.18 ± 0.03 |
| **Uterus [%]** | - | - | | - | 0.21 ± 0.03 | | 0.19 ± 0.03 | | 0.20 ± 0.04 |
| **Ovary (right) [%]** | - | - | | - | 0.02 ± 0.00 | | 0.02 ± 0.00 | | 0.02 ± 0.00 |
| **Ovary (left) [%]** | - | - | | - | 0.02 ± 0.00 | | 0.02 ± 0.00 | | 0.02 ± 0.00 |
| **Testis (right) [%]** | 0.43 ± 0.03 | 0.44 ± 0.03 | | 0.41 ± 0.03 | - | | - | | - |
| **Testis (left) [%]** | 0.43 ± 0.03 | 0.44 ± 0.03 | | 0.41 ± 0.03 | - | | - | | - |
| **Epididymis (right) [%]** | 0.14 ± 0.01 | 0.15 ± 0.01 | | 0.14 ± 0.01 | - | | - | | - |
| **Epididymis (left) [%]** | 0.15 ± 0.01 | 0.15 ± 0.01 | | 0.14 ± 0.02 | - | | - | | - |
| **Brain [%]** | 0.51 ± 0.02 | 0.53 ± 0.04 | | 0.51 ± 0.03 | 0.84 ± 0.06 | | 0.80 ± 0.03 | | 0.82 ± 0.04 |

| **haematology** | **male** | | | **female** | | |
| --- | --- | --- | --- | --- | --- | --- |
|  | **control** | **11% GMO** | **33% GMO** | **control** | **11% GMO** | **33% GMO** |
| **WBC (10³/μl)** | 8.98 ± 1.62 | 11.37 ± 3.23 | 11.32 ± 1.66 | 6.52 ± 1.94 | 10.76 ± 2.45 * | 10.73 ± 1.65 * |
| **RBC (10^6^/μl)** | 8.21 ± 0.21 | 8.27 ± 0.08 | 8.03 ± 0.32 | 7.59 ± 0.23 | 7.73 ± 0.17 | 7.49 ± 0.22 |
| **HGB (g/dl)** | 15.85 ± 0.54 | 16.49 ± 0.28 | 16.01 ± 0.32 | 15.52 ± 0.39 | 15.81 ± 0.34 | 15.59 ± 0.22 |
| **HCT (%)** | 44.38 ± 1.21 | 45.74 ± 0.71 | 44.19 ± 0.73 | 43.47 ± 1.37 | 43.78 ± 0.75 | 42.75 ± 0.69 |
| **MCV (fl)** | 54.05 ± 0.20 | 55.34 ± 0.67 * | 55.13 ± 1.54 | 57.27 ± 0.36 | 56.65 ± 1.36 | 57.11 ± 1.64 |
| **MCH (pg)** | 19.31 ± 0.43 | 19.97 ± 0.19 * | 19.97 ± 0.78 | 20.47 ± 0.15 | 20.44 ± 0.67 | 20.84 ± 0.47 |
| **MCHC (g/dl)** | 35.71 ± 0.71 | 36.06 ± 0.19 | 36.24 ± 0.47 | 35.73 ± 0.40 | 36.12 ± 0.46 | 36.48 ± 0.35 * |
| **PLT (10³/μl)** | 783.10 ± 70.37 | 710.00 ± 210.96 | 736.70 ± 88.54 | 708.00 ± 168.43 | 610.10 ± 111.46 | 672.80 ± 112.14 |
| **LYM (10^3^/µl)** | 6.66 ± 1.10 | 8.50 ± 2.28 | 8.31 ± 1.28 | 4.28 ± 1.18 | 5.78 ± 1.16 | 6.43 ± 0.94 * |
| **leucocyte counts** |  | | | | | |
| **Lymphocytes [%]** | 74.80 ± 3.31 | 76.00 ± 3.82 | 79.20 ± 3.63 | 69.50 ± 4.53 | 64.50 ± 6.28 | 70.30 ± 5.92 |
| **Neutrophils [%]** | 20.80 ± 3.56 | 18.00 ± 4.24 | 15.70 ± 2.36 * | 26.90 ± 4.60 | 31.45 ± 6.22 | 26.50 ± 5.30 |
| **Monocytes [%]** | 2.90 ± 1.08 | 2.95 ± 0.45 | 2.80 ± 0.65 | 2.50 ± 0.87 | 1.90 ± 0.42 | 1.80 ± 0.67 |
| **Eosinohils [%]** | 1.50 ± 0.47 | 3.05 ± 1.23 * | 2.30 ± 1.02 | 1.10 ± 0.74 | 2.15 ± 0.99 | 1.35 ± 0.49 |
| **Basophils [%]** | 0.00 ± 0.00 | 0.00 ± 0.00 | 0.00 ± 0.00 | 0.00 ± 0.00 | 0.00 ± 0.00 | 0.05 ± 0.11 |

| **clinical biochemistry** | **male** | | | | | **female** | | | | | |
| --- | --- | --- | --- | --- | --- | --- | --- | --- | --- | --- | --- |
|  | **control** | **11% GMO** | | **33% GMO** | | **control** | | **11% GMO** | | **33% GMO** | |
| **ALP (µkat/l)** | 1.51 ± 0.22 | | 1.09 ± 0.24 * | | 1.24 ± 0.21 | | 0.70 ± 0.14 | | 0.62 ± 0.11 | | 0.74 ± 0.16 |
| **ALT (µkat/l)** | 0.49 ± 0.04 | | 0.46 ± 0.02 | | 0.52 ± 0.07 | | 0.43 ± 0.07 | | 0.63 ± 0.15 * | | 0.77 ± 0.11 * |
| **AST (µkat/l)** | 2.36 ± 0.38 | | 2.56 ± 0.67 | | 2.81 ± 0.44 | | 2.61 ± 0.45 | | 4.14 ± 1.10 * | | 4.47 ± 0.89 * |
| **ALB (g/l)** | 38.53 ± 1.71 | | 37.94 ± 1.01 | | 37.63 ± 2.13 | | 46.80 ± 3.37 | | 47.19 ± 1.91 | | 45.60 ± 2.94 |
| **GLU (mmol/l)** | 5.50 ± 0.48 | | 5.72 ± 0.61 | | 5.33 ± 0.50 | | 5.38 ± 0.69 | | 5.47 ± 1.16 | | 5.25 ± 0.56 |
| **CREA (µmol/l)** | 44.62 ± 7.59 | | 43.91 ± 6.61 | | 44.22 ± 3.10 | | 45.43 ± 2.36 | | 45.14 ± 6.46 | | 44.69 ± 4.68 |
| **TP (g/l)** | 63.46 ± 2.64 | | 61.56 ± 1.50 | | 62.20 ± 2.48 | | 73.67 ± 4.93 | | 74.12 ± 2.59 | | 72.63 ± 4.10 |
| **U (mmol/l)** | 5.34 ± 0.40 | | 5.30 ± 0.13 | | 4.80 ± 0.22^c^ | | 5.01 ± 0.23 | | 5.49 ± 0.25^c^ | | 5.14 ± 0.33 |
| **CHOL (mmol/l)** | 2.15 ± 0.30 | | 2.07 ± 0.13 | | 2.22 ± 0.20 | | 2.04 ± 0.30 | | 1.99 ± 0.21 | | 1.83 ± 0.29 |
| **Ca (mmol/l)** | 2.47 ± 0.04 | | 2.45 ± 0.05 | | 2.51 ± 0.04 | | 2.56 ± 0.05 | | 2.58 ± 0.05 | | 2.59 ± 0.05 |
| **Cl (mmol/l)** | 102.80 ± 1.25 | | 103.20 ± 1.52 | | 101.20 ± 0.84 | | 101.50 ± 1.50 | | 99.80 ± 1.25 | | 100.40 ± 1.14 |
| **K (mmol/l)** | 5.09 ± 0.32 | | 4.82 ± 0.24 | | 5.44 ± 0.44 | | 4.41 ± 0.30 | | 4.63 ± 0.16 | | 4.96 ± 0.47 |
| **Na (mmol/l)** | 142.50 ± 2.35 | | 142.30 ± 1.99 | | 142.10 ± 1.02 | | 143.30 ± 1.44 | | 143.90 ± 1.34 | | 143.80 ± 1.68 |
| **P (mmol/l)** | 2.38 ± 0.15 | | 2.48 ± 0.10 | | 2.56 ± 0.20 | | 1.71 ± 0.12 | | 2.03 ± 0.32 | | 2.25 ± 0.47 |
| **TRG (mmol/l)** | 0.80 ± 0.17 | | 0.74 ± 0.13 | | 0.87 ± 0.23 | | 0.63 ± 0.09 | | 0.74 ± 0.04 | | 0.85 ± 0.05 * |

| **feed consumption** | **male** | | | **female** | | |
| --- | --- | --- | --- | --- | --- | --- |
|  | **control** | **11% GMO** | **33% GMO** | **control** | **11% GMO** | **33% GMO** |
| **Week 0 [g]** | 219.17 ± 16.83 | 213.74 ± 3.61 | 229.09 ± 6.88 | 166.16 ± 15.93 | 159.45 ± 6.35 | 173.83 ± 7.18 |
| **Week 1 [g]** | 311.18 ± 5.94 | 294.19 ± 10.61 | 328.71 ± 8.20 | 224.16 ± 12.46 | 234.73 ± 16.20 | 233.07 ± 9.19 |
| **Week 2 [g]** | 310.15 ± 10.17 | 284.37 ± 20.48 | 306.31 ± 19.22 | 196.30 ± 31.32 | 209.05 ± 8.32 | 219.54 ± 14.14 |
| **Week 3 [g]** | 260.84 ± 11.80 | 239.50 ± 19.28 | 254.53 ± 14.27 | 186.14 ± 18.58 | 180.61 ± 7.88 | 186.85 ± 7.97 |
| **Week 4 [g]** | 324.60 ± 17.27 | 304.18 ± 28.75 | 308.37 ± 20.12 | 228.18 ± 14.69 | 234.67 ± 13.88 | 237.86 ± 11.81 |
| **Week 5 [g]** | 308.56 ± 23.16 | 280.51 ± 23.64 | 297.20 ± 16.19 | 217.43 ± 18.99 | 213.02 ± 7.95 | 225.64 ± 12.35 |
| **Week 6 [g]** | 298.94 ± 20.51 | 275.36 ± 24.56 | 290.88 ± 14.29 | 205.66 ± 16.87 | 209.99 ± 6.94 | 216.97 ± 20.43 |
| **Week 7 [g]** | 255.45 ± 14.09 | 239.09 ± 17.83 | 257.97 ± 6.10 | 181.69 ± 13.38 | 177.05 ± 10.41 | 184.28 ± 12.77 |
| **Week 8 [g]** | 315.96 ± 13.10 | 295.36 ± 24.12 | 315.30 ± 9.04 | 219.30 ± 16.85 | 212.93 ± 22.83 | 215.64 ± 12.22 |
| **Week 9 [g]** | 301.80 ± 16.68 | 273.46 ± 22.54 | 304.47 ± 9.33 | 218.30 ± 27.36 | 219.40 ± 14.92 | 226.43 ± 23.61 |
| **Week 10 [g]** | 305.85 ± 16.17 | 272.33 ± 23.89 | 296.37 ± 11.66 | 205.63 ± 21.03 | 207.06 ± 6.27 | 212.70 ± 16.89 |
| **Week 11 [g]** | 302.92 ± 16.33 | 273.05 ± 25.11 | 294.52 ± 15.24 | 201.00 ± 22.04 | 206.76 ± 11.81 | 211.08 ± 11.85 |
| **Week 12 [g]** | 261.50 ± 9.58 | 230.43 ± 21.15 | 259.13 ± 13.45 | 173.64 ± 22.58 | 177.31 ± 4.57 | 184.05 ± 10.97 |

| **feed efficiency** | **male** | | | **female** | | |
| --- | --- | --- | --- | --- | --- | --- |
|  | **control** | **11% GMO** | **33% GMO** | **control** | **11% GMO** | **33% GMO** |
| **Week 1 [%]** | 11.95 ± 2.16 | 18.02 ± 1.51 | 12.86 ± 1.76 | 10.07 ± 2.47 | 11.95 ± 2.32 | 9.42 ± 0.71 |
| **Week 2 [%]** | 14.37 ± 1.13 | 15.51 ± 1.76 | 13.55 ± 0.93 | 8.57 ± 1.28 | 9.39 ± 2.15 | 8.30 ± 2.48 |
| **Week 3 [%]** | 10.76 ±0.79 | 11.94 ± 2.35 | 11.47 ± 1.46 | 3.22 ± 11.52 | 8.82 ± 1.65 | 8.73 ± 0.71 |
| **Week 4 [%]** | 8.43 ± 1.42 | 8.48 ± 0.83 | 9.27 ± 1.97 | 5.86 ± 8.25 | 3.52 ± 1.14 | 2.79 ± 1.43 |
| **Week 5 [%]** | 9.74 ± 0.90 | 8.87 ± 0.92 | 8.00 ± 2.03 | 5.25 ± 1.36 | 4.79 ± 1.60 | 4.82 ± 0.60 |
| **Week 6 [%]** | 6.80 ± 0.70 | 6.20 ± 1.16 | 7.35 ± 0.70 | 5.86 ± 3.17 | 5.17 ± 2.01 | 5.12 ± 1.20 |
| **Week 7 [%]** | 6.76 ± 1.05 | 7.30 ± 0.59 | 7.34 ± 0.61 | 4.17 ± 1.74 | 4.36 ± 1.16 | 4.16 ± 1.61 |
| **Week 8 [%]** | 3.95 ± 1.05 | 3.85 ± 1.14 | 2.84 ± 4.46 | -0.96 ± 1.29 | 0.03 ± 0.78 | -0.60 ± 2.16 |
| **Week 9 [%]** | 5.11 ± 1.66 | 5.34 ± 0.56 | 5.61 ± 0.74 | 2.28 ± 1.07 | 2.70 ± 0.46 | 1.75 ± 0.92 |
| **Week 10 [%]** | 5.20 ± 0.94 | 4.46 ± 1.09 | 5.81 ± 1.10 | 2.41 ± 2.48 | 1.32 ± 1.06 | 2.33 ± 1.11 |
| **Week 11 [%]** | 4.53 ± 0.67 | 3.50 ± 1.43 | 4.14 ± 1.36 | 2.67 ± 1.27 | 3.50 ± 1.24 | 2.67 ± 1.16 |
| **Week 12 [%]** | 3.95 ± 0.96 | 3.81 ± 1.11 | 4.59 ± 1.00 | 1.45 ± 1.40 | 3.24 ± 1.94 | 1.98 ± 1.65 |
| **Week 13 [%]** | 2.37 ± 0.23 | 0.57 ± 1.83 | 2.36 ± 1.32 | 0.04 ± 2.73 | -0.70 ± 1.86 | 0.06 ± 2.45 |

| **urinalysis** | **male** | | | **female** | | |
| --- | --- | --- | --- | --- | --- | --- |
|  | **control** | **11% GMO** | **33% GMO** | **control** | **11% GMO** | **33% GMO** |
| **N** | **10** | **10** | **10** | **10** | **10** | **10** |
| **BIL-U (mmol/l)**  negative | 10 | 10 | 10 | 10 | 10 | 10 |
| **LEU (Leu/µl)**  negative  25 leu/µg  100 leu/µg  500 leu/µg | 10 | 10 | 10 | 7  3 | 4  6 | 3  6  1 |
| **NIT (+/-)**  negative  positive | 10 | 10 | 10 | 10 | 10 | 10 |
| **PROT (g/l)**  negative  0,25 g/l  0.75 g/l  1,5 g/l | 10 | 9  1 | 7  3 | 10 | 10 | 10 |
| **GLU-U (mmol/l)**  normal | 10 | 10 | 10 | 10 | 10 | 10 |
| **HEM (Ery/µl)**  negative  10 ery/µg  25 ery/µg  50 ery/µg  250 ery/µg | 9  1 | 8  1  1 | 9  1 | 10 | 10 | 10 |
| **KET (mmol/l)**  negative  0,5 mmol/l  1,5 mmol/l  5 mmol/l | 3  3  4 | 1  1  8 | 1  2  5  2 | 7  2  1 | 3  3  4 | 5  3  2 |
| **pH**  5,0  6,0  6,5  7,0 | 3  7 | 5  5 | 3  7 | 8  1  1 | 9  1 | 4  6 |
| **URO (mmol/l)**  normal | 10 | 10 | 10 | 10 | 10 | 10 |
| **OSM (moms)**  Median  Mean  SD  Min  Max | 360.5  387.9  94.2  300.0  585.0 | 472.5  451.2  111.8  277.0  668.0 | 362.0  400.6  171.2  229.0  883.0 | 477.5  461.1  107.2  301.0  658.0 | 506.0  531.6  188.6  278.0  798.0 | 631.5  605.4  184.0  413.0  966.0 |

***** confidence interval of SES to control does not include the zero value.

NOTE: individual significances (based on 95% confidence intervals of SES) for body weight, feed consumption and feed efficiency week-by-week have not been calculated
