# Supplementary material for: Variability of control data and relevance of observed group differences in five oral toxicity studies with genetically modified maize MON810 in rats
Source: Arch Toxicol. 2016 Oct 11;91(4):1977–2006. doi: 10.1007/s00204-016-1857-x (PMC5364247; doi:10.1007/s00204-016-1857-x)
Supplement: Supplementary file 2 — Supplementary material 2 (DOCX 34 kb) [file 204_2016_1857_MOESM2_ESM.docx]

**ESM-Table 2:** Trial E: mean ± standard deviations of body weight week-by-week, relative organ weights, haematology and clinical biochemistry parameters (significant groups differences of 11% GMO and 33% GMO to control are marked) as well as feed consumption and efficiency week-by-week

| **body weight** | **male** | | | **female** | | |
| --- | --- | --- | --- | --- | --- | --- |
|  | **control** | **11% GMO** | **33% GMO** | **control** | **11% GMO** | **33% GMO** |
| **Week 0 [g]** | 158.05 ± 4.98 | 159.30 ± 3.84 | 161.10 ± 3.29 | 142.08 ± 4.59 | 143.57 ± 4.12 | 144.48 ± 3.88 |
| **Week 1 [g]** | 187.75 ± 5.91 | 188.64 ± 1.79 | 190.05 ± 1.05 | 156.39 ± 7.48 | 161.61 ± 5.71 | 161.29 ± 4.33 |
| **Week 2 [g]** | 231.00 ± 8.00 | 230.58 ± 3.82 | 232.64 ± 3.10 | 176.24 ± 12.00 | 180.01 ± 6.12 | 179.00 ± 5.29 |
| **Week 3 [g]** | 260.14 ± 8.83 | 262.12 ± 6.41 | 264.01 ± 4.74 | 193.62 ± 12.07 | 194.79 ± 7.33 | 192.62 ± 5.40 |
| **Week 4 [g]** | 278.93 ± 11.47 | 282.41 ± 8.87 | 284.16 ± 8.51 | 201.12 ± 12.27 | 202.38 ± 8.78 | 199.13 ± 6.53 |
| **Week 5 [g]** | 305.32 ± 12.02 | 307.36 ± 10.51 | 311.37 ± 8.66 | 212.66 ± 14.61 | 213.75 ± 10.68 | 210.51 ± 9.22 |
| **Week 6 [g]** | 323.66 ± 12.98 | 325.41 ± 12.91 | 318.71 ± 26.89 | 222.00 ± 19.16 | 222.31 ± 12.06 | 220.02 ± 9.45 |
| **Week 7 [g]** | 344.66 ± 15.30 | 342.50 ± 13.65 | 346.59 ± 13.40 | 228.83 ± 17.80 | 229.87 ± 12.70 | 228.93 ± 8.88 |
| **Week 8 [g]** | 352.02 ± 16.44 | 351.60 ± 15.76 | 356.02 ± 16.58 | 231.74 ± 19.64 | 229.20 ± 12.22 | 229.85 ± 8.83 |
| **Week 9 [g]** | 367.70 ± 13.45 | 364.90 ± 15.18 | 371.39 ± 18.75 | 236.63 ± 21.33 | 235.92 ± 14.25 | 235.87 ± 11.57 |
| **Week 10 [g]** | 381.60 ± 13.24 | 378.16 ± 17.97 | 383.69 ± 19.05 | 240.22 ± 23.16 | 237.89 ± 13.33 | 237.15 ± 10.01 |
| **Week 11 [g]** | 390.49 ± 14.37 | 388.61 ± 18.81 | 394.85 ± 20.06 | 243.07 ± 21.48 | 242.59 ± 13.84 | 241.39 ± 7.53 |
| **Week 12 [g]** | 401.20 ± 11.42 | 399.55 ± 19.79 | 405.78 ± 22.28 | 246.96 ± 20.95 | 246.64 ± 15.80 | 246.74 ± 10.20 |
| **Week 13 [g]** | 404.74 ± 11.23 | 403.45 ± 23.01 | 409.79 ± 21.90 | 250.15 ± 25.27 | 248.10 ± 14.42 | 247.77 ± 8.62 |

| **organ weights** | **male** | | | | | **female** | | | | | |
| --- | --- | --- | --- | --- | --- | --- | --- | --- | --- | --- | --- |
|  | **33% GMO** | **control** | | **33% GMO** | | **control** | | **33% GMO** | | **control** | |
| **Kidney (right) [%]** | 0.28 ± 0.02 | | 0.28 ± 0.01 | | 0.28 ± 0.01 | | 0.30 ± 0.02 | | 0.29 ± 0.02 | | 0.30 ± 0.02 |
| **Kidney (left) [%]** | 0.27 ± 0.02 | | 0.29 ± 0.01 | | 0.27 ± 0.00 | | 0.29 ± 0.02 | | 0.29 ± 0.02 | | 0.30 ± 0.03 |
| **Spleen [%]** | 0.17 ± 0.01 | | 0.18 ± 0.01 | | 0.18 ± 0.02 | | 0.21 ± 0.02 | | 0.22 ± 0.01 | | 0.22 ± 0.02 |
| **Liver [%]** | 2.13 ± 0.06 | | 2.15 ± 0.11 | | 2.12 ± 0.12 | | 2.47 ± 0.13 | | 2.49 ± 0.02 | | 2.34 ± 0.12 |
| **Adrenal (right) [%]** | 0.01 ± 0.00 | | 0.01 ± 0.00 | | 0.01 ± 0.00 | | 0.01 ± 0.00 | | 0.01 ± 0.00 | | 0.01 ± 0.00 |
| **Adrenal (left) [%]** | 0.01 ± 0.00 | | 0.01 ± 0.00 | | 0.01 ± 0.00 | | 0.01 ± 0.00 | | 0.02 ± 0.00 | | 0.02 ± 0.00 |
| **Lung [%]** | 0.30 ± 0.02 | | 0.32 ± 0.01 | | 0.30 ± 0.01 | | 0.40 ± 0.03 | | 0.41 ± 0.02 | | 0.41 ± 0.02 |
| **Heart [%]** | 0.23 ± 0.01 | | 0.23 ± 0.02 | | 0.24 ± 0.01 | | 0.27 ± 0.02 | | 0.28 ± 0.01 | | 0.28 ± 0.01 |
| **Thymus [%]** | 0.10 ± 0.02 | | 0.10 ± 0.02 | | 0.09 ± 0.01 | | 0.13 ± 0.01 | | 0.11 ± 0.02 | | 0.14 ± 0.02 |
| **Pancreas [%]** | 0.14 ± 0.02 | | 0.14 ± 0.02 | | 0.15 ± 0.03 | | 0.17 ± 0.01 | | 0.18 ± 0.02 | | 0.19 ± 0.06 |
| **Uterus [%]** | - | | - | | - | | 0.19 ± 0.02 | | 0.18 ± 0.03 | | 0.17 ± 0.03 |
| **Ovary (right) [%]** | - | | - | | - | | 0.02 ± 0.00 | | 0.02 ± 0.00 | | 0.02 ± 0.00 |
| **Ovary (left) [%]** | - | | - | | - | | 0.02 ± 0.00 | | 0.02 ± 0.00 | | 0.02 ± 0.00 |
| **Testis (right) [%]** | 0.46 ± 0.01 | | 0.47 ± 0.05 | | 0.45 ± 0.02 | | - | | - | | - |
| **Testis (left) [%]** | 0.46 ± 0.02 | | 0.47 ± 0.05 | | 0.45 ± 0.02 | | - | | - | | - |
| **Epididymis (right) [%]** | 0.15 ± 0.01 | | 0.17 ± 0.02 | | 0.15 ± 0.01 | | - | | - | | - |
| **Epididymis (left) [%]** | 0.15 ± 0.01 | | 0.16 ± 0.02 | | 0.15 ± 0.01 | | - | | - | | - |
| **Brain [%]** | 0.55 ± 0.02 | | 0.55 ± 0.03 | | 0.55 ± 0.03 | | 0.84 ± 0.08 | | 0.84 ± 0.05 | | 0.83 ± 0.02 |

| **haematology** | **male** | | | **female** | | |
| --- | --- | --- | --- | --- | --- | --- |
|  | **control** | **11% GMO** | **33% GMO** | **control** | **11% GMO** | **33% GMO** |
| **WBC (10³/μl)** | 8.12 ± 0.81 | 10.67 ± 2.22 | 9.39 ± 1.00 | 9.11 ± 0.79 | 8.27 ± 1.49 | 7.42 ± 0.89 * |
| **RBC (10^6^/μl)** | 8.20 ± 0.14 | 8.06 ± 0.34 | 8.22 ± 0.25 | 7.87 ± 0.31 | 7.54 ± 0.22 | 7.71 ± 0.34 |
| **HGB (g/dl)** | 16.16 ± 0.20 | 15.86 ± 0.71 | 16.33 ± 0.21 | 15.69 ± 0.32 | 15.51 ± 0.50 | 15.74 ± 0.70 |
| **HCT (%)** | 45.29 ± 0.68 | 44.26 ± 1.86 | 45.29 ± 0.58 | 44.52 ± 1.02 | 42.76 ± 1.38 | 43.28 ± 1.90 |
| **MCV (fl)** | 55.26 ± 0.42 | 54.96 ± 1.80 | 55.15 ± 1.41 | 56.65 ± 0.99 | 56.75 ± 0.92 | 56.14 ± 0.39 |
| **MCH (pg)** | 19.71 ± 0.41 | 19.71 ± 0.75 | 19.89 ± 0.82 | 19.97 ± 0.62 | 20.58 ± 0.28 | 20.44 ± 0.38 |
| **MCHC (g/dl)** | 35.69 ± 0.57 | 35.85 ± 0.53 | 36.07 ± 0.62 | 35.26 ± 0.68 | 36.30 ± 0.81 | 36.41 ± 0.74 |
| **PLT (10³/μl)** | 762.50 ± 16.46 | 706.40 ± 85.16 | 772.30 ± 70.62 | 740.40 ± 95.85 | 770.50 ± 96.07 | 648.00 ± 141.50 |
| **LYM (10^3^/µl)** | 5.99 ± 0.57 | 7.15 ± 1.15 | 6.82 ± 0.46 | 5.50 ± 0.88 | 4.84 ± 0.44 | 5.01 ± 0.84 |
| **leucocyte counts** |  | | | | | |
| **Lymphocytes [%]** | 72.50 ± 3.06 | 78.30 ± 4.37 | 77.50 ± 0.71 * | 73.70 ± 5.31 | 65.10 ± 4.08 * | 74.90 ± 3.17 |
| **Neutrophils [%]** | 23.80 ± 2.73 | 17.55 ± 3.62 * | 18.50 ± 1.46 * | 21.50 ± 3.89 | 31.30 ± 4.37 * | 21.70 ± 3.51 |
| **Monocytes [%]** | 1.90 ± 0.89 | 2.10 ± 0.65 | 1.95 ± 0.72 | 3.20 ± 1.01 | 1.70 ± 0.97 | 1.70 ± 0.57 * |
| **Eosinohils [%]** | 1.80 ± 0.97 | 2.05 ± 1.30 | 2.05 ± 1.15 | 1.55 ± 0.97 | 1.90 ± 0.96 | 1.70 ± 0.76 |
| **Basophils [%]** | 0.00 ± 0.00 | 0.00 ± 0.00 | 0.00 ± 0.00 | 0.05 ± 0.11 | 0.00 ± 0.00 | 0.00 ± 0.00 |

| **organ weights** | **male** | | | | | **female** | | | | |
| --- | --- | --- | --- | --- | --- | --- | --- | --- | --- | --- |
|  | **33% GMO** | **control** | | **33% GMO** | | **control** | **33% GMO** | | **control** | |
| **ALP (µkat/l)** | 1.48 ± 0.27 | | 1.25 ± 0.11 | | 1.36 ± 0.09 | 0.47 ± 0.14 | | 0.66 ± 0.14 | | 0.55 ± 0.13 |
| **ALT (µkat/l)** | 0.46 ± 0.04 | | 0.49 ± 0.08 | | 0.50 ± 0.03 | 0.51 ± 0.17 | | 0.61 ± 0.13 | | 0.47 ± 0.13 |
| **AST (µkat/l)** | 2.24 ± 0.19 | | 2.33 ± 0.47 | | 2.28 ± 0.17 | 2.97 ± 0.40 | | 3.12 ± 0.32 | | 2.54 ± 0.30 |
| **ALB (g/l)** | 38.14 ± 1.29 | | 37.34 ± 1.58 | | 38.78 ± 0.98 | 46.76 ± 2.02 | | 47.18 ± 1.67 | | 46.31 ± 2.19 |
| **GLU (mmol/l)** | 6.18 ± 0.94 | | 5.87 ± 0.33 | | 5.30 ± 0.59 | 5.86 ± 0.43 | | 5.43 ± 0.55 | | 5.33 ± 0.64 |
| **CREA (µmol/l)** | 42.77 ± 3.22 | | 45.91 ± 3.90 | | 46.25 ± 3.87 | 47.38 ± 5.26 | | 45.93 ± 3.66 | | 46.44 ± 5.33 |
| **TP (g/l)** | 63.13 ± 1.54 | | 61.93 ± 1.03 | | 63.30 ± 0.88 | 74.94 ± 1.78 | | 76.34 ± 1.58 | | 74.17 ± 2.58 |
| **U (mmol/l)** | 5.35 ± 0.43 | | 5.52 ± 0.35 | | 5.18 ± 0.28 | 5.52 ± 0.38 | | 5.43 ± 0.32 | | 5.80 ± 0.32 |
| **CHOL (mmol/l)** | 2.22 ± 0.15 | | 1.90 ± 0.28 | | 1.97 ± 0.24 | 1.79 ± 0.25 | | 1.99 ± 0.05 | | 1.72 ± 0.19 |
| **Ca (mmol/l)** | 2.60 ± 0.24 | | 2.48 ± 0.07 | | 2.45 ± 0.02 | 2.57 ± 0.04 | | 2.59 ± 0.04 | | 2.56 ± 0.05 |
| **Cl (mmol/l)** | 102.40 ± 1.47 | | 103.40 ± 2.22 | | 102.30 ± 0.57 | 101.70 ± 1.35 | | 100.70 ± 1.35 | | 102.70 ± 0.91 |
| **K (mmol/l)** | 5.08 ± 0.45 | | 4.92 ± 0.14 | | 4.93 ± 0.34 | 4.92 ± 0.63 | | 4.99 ± 0.53 | | 5.10 ± 0.55 |
| **Na (mmol/l)** | 141.40 ± 2.95 | | 142.20 ± 1.15 | | 141.50 ± 1.37 | 141.50 ± 2.32 | | 142.30 ± 0.84 | | 142.20 ± 1.60 |
| **P (mmol/l)** | 2.43 ± 0.22 | | 2.48 ± 0.21 | | 2.27 ± 0.21 | 1.94 ± 0.43 | | 1.85 ± 0.25 | | 1.91 ± 0.21 |
| **TRG (mmol/l)** | 0.82 ± 0.19 | | 0.77 ± 0.14 | | 0.84 ± 0.10 | 0.70 ± 0.05 | | 0.70 ± 0.16 | | 0.71 ± 0.05 |

| **feed consumption** | **male** | | | **female** | | |
| --- | --- | --- | --- | --- | --- | --- |
|  | **control** | **11% GMO** | **33% GMO** | **control** | **11% GMO** | **33% GMO** |
| **Week 1 [g]** | 207.35 ± 11.69 | 203.72 ± 11.55 | 202.99 ± 7.63 | 151.52 ± 12.83 | 158.54 ± 11.14 | 146.79 ± 6.50 |
| **Week 2 [g]** | 281.54 ± 36.32 | 292.25 ± 15.24 | 300.75 ± 11.78 | 217.90 ± 21.31 | 227.52 ± 16.76 | 217.86 ± 17.42 |
| **Week 3 [g]** | 273.02 ± 25.43 | 276.84 ± 19.35 | 274.83 ± 18.46 | 210.81 ± 14.55 | 205.70 ± 13.26 | 192.77 ± 10.99 |
| **Week 4 [g]** | 241.00 ± 11.30 | 234.52 ± 13.68 | 232.37 ± 14.29 | 181.13 ± 18.81 | 179.07 ± 10.19 | 165.76 ± 8.82 |
| **Week 5 [g]** | 297.89 ± 7.70 | 291.67 ± 13.63 | 294.25 ± 13.21 | 228.44 ± 20.90 | 222.24 ± 16.92 | 208.29 ± 11.84 |
| **Week 6 [g]** | 279.46 ± 14.07 | 271.83 ± 17.90 | 257.87 ± 37.06 | 216.08 ± 19.49 | 208.70 ± 20.42 | 196.12 ± 11.14 |
| **Week 7 [g]** | 269.28 ± 12.03 | 263.75 ± 11.99 | 273.25 ± 25.22 | 212.76 ± 20.37 | 201.00 ± 17.27 | 196.42 ± 12.84 |
| **Week 8 [g]** | 232.63 ± 10.88 | 228.51 ± 14.28 | 228.32 ± 13.49 | 184.66 ± 27.08 | 174.60 ± 15.93 | 170.21 ± 10.47 |
| **Week 9 [g]** | 287.23 ± 9.13 | 287.23 ± 14.64 | 288.99 ± 15.59 | 214.43 ± 21.86 | 212.72 ± 17.98 | 203.89 ± 14.68 |
| **Week 10 [g]** | 275.44 ± 7.52 | 268.21 ± 17.15 | 265.54 ± 17.08 | 203.54 ± 19.69 | 199.80 ± 24.85 | 193.82 ± 14.42 |
| **Week 11 [g]** | 276.40 ± 8.27 | 266.95 ± 18.06 | 266.83 ± 17.46 | 199.26 ± 16.39 | 202.51 ± 19.57 | 189.00 ± 9.92 |
| **Week 12 [g]** | 269.92 ± 6.06 | 264.46 ± 22.74 | 262.94 ± 13.51 | 197.50 ± 19.26 | 194.18 ± 16.51 | 185.77 ± 11.55 |
| **Week 13 [g]** | 236.81 ± 4.76 | 230.47 ± 18.41 | 226.87 ± 13.72 | 171.68 ± 18.01 | 170.83 ± 17.33 | 164.81 ± 12.06 |

| **feed efficiency** | **male** | | | **female** | | |
| --- | --- | --- | --- | --- | --- | --- |
|  | **control** | **11% GMO** | **33% GMO** | **control** | **11% GMO** | **33% GMO** |
| **Week 1 [%]** | 14.34 ± 1.22 | 14.48 ± 2.27 | 14.29 ± 1.72 | 9.33 ± 2.00 | 11.26 ± 2.96 | 11.46 ± 1.34 |
| **Week 2 [%]** | 15.48 ± 1.61 | 14.33 ± 0.99 | 14.15 ± 0.45 | 9.01 ± 1.78 | 8.10 ± 1.09 | 8.15 ± 1.20 |
| **Week 3 [%]** | 10.77 ± 1.82 | 11.40 ± 0.90 | 11.44 ± 0.96 | 8.24 ± 1.34 | 7.17 ± 0.64 | 7.08 ± 0.92 |
| **Week 4 [%]** | 7.75 ± 1.51 | 8.62 ± 0.95 | 8.60 ± 1.55 | 4.16 ± 0.58 | 4.21 ± 1.00 | 3.90 ± 1.40 |
| **Week 5 [%]** | 8.85 ± 1.70 | 8.55 ± 0.61 | 9.26 ± 0.58 | 4.99 ± 1.56 | 5.10 ± 0.82 | 5.39 ± 1.75 |
| **Week 6 [%]** | 6.55 ± 0.78 | 6.62 ± 0.95 | 1.59 ± 11.63 | 4.20 ± 1.89 | 4.01 ± 1.72 | 4.85 ± 1.57 |
| **Week 7 [%]** | 7.79 ± 0.86 | 6.48 ± 0.65 | 9.76 ± 6.67 | 3.29 ± 1.60 | 3.75 ± 1.62 | 4.54 ± 1.77 |
| **Week 8 [%]** | 3.14 ± 1.36 | 3.95 ± 1.29 | 4.10 ± 1.66 | 1.43 ± 2.38 | -0.42 ± 1.98 | 0.53 ± 0.67 |
| **Week 9 [%]** | 5.46 ± 1.84 | 4.63 ± 1.55 | 5.32 ± 1.22 | 2.23 ± 1.02 | 3.12 ± 1.41 | 2.88 ± 1.61 |
| **Week 10 [%]** | 5.06 ± 0.58 | 4.90 ± 0.76 | 4.64 ± 0.45 | 1.68 ± 1.99 | 1.05 ± 1.12 | 0.73 ± 1.41 |
| **Week 11 [%]** | 3.19 ± 1.53 | 3.92 ± 1.14 | 4.19 ± 0.73 | 1.49 ± 1.68 | 2.29 ± 1.12 | 2.30 ± 1.50 |
| **Week 12 [%]** | 3.99 ± 1.67 | 4.14 ± 0.24 | 4.15 ± 1.05 | 2.01 ± 1.38 | 2.06 ± 1.90 | 2.84 ± 1.96 |
| **Week 13 [%]** | 1.49 ± 1.34 | 1.62 ± 1.37 | 1.74 ± 1.69 | 1.59 ± 3.71 | 0.88 ± 2.59 | 0.64 ± 1.79 |

| **urinalysis** | **male** | | | **female** | | |
| --- | --- | --- | --- | --- | --- | --- |
|  | **control** | **11% GMO** | **33% GMO** | **control** | **11% GMO** | **33% GMO** |
| **N** | **10** | **10** | **10** | **10** | **10** | **10** |
| **BIL-U (mmol/l)**  negative | 10 | 10 | 10 | 10 | 10 | 10 |
| **LEU (Leu/µl)**  negative  25 leu/µg  100 leu/µg  500 leu/µg | 10 | 10 | 9  1 | 9  1 | 10 | 7  2  1 |
| **NIT (+/-)**  negative  positive | 10 | 10 | 9  1 | 10 | 10 | 10 |
| **PROT (g/l)**  negative  0,25 g/l  0.75 g/l  1,5 g/l | 9  1 | 10 | 8  1  1 | 10 | 10 | 10 |
| **GLU-U (mmol/l)**  normal | 10 | 10 | 10 | 10 | 10 | 10 |
| **HEM (Ery/µl)**  negative  10 ery/µg  25 ery/µg  50 ery/µg  250 ery/µg | 10 | 9  1 | 9  1 | 10 | 10 | 10 |
| **KET (mmol/l)**  negative  0.25 mmol/l  0,5 mmol/l  1,5 mmol/l  5 mmol/l | 2  8 | 2  2  6 | 1  1  8 | 9  1 | 8  2 | 6  4 |
| **pH**  5,0  6,0  6,5  7,0  8,0 | 1  2  1  4  2 | 1  1  7  1 | 3  7 | 10 | 8  1  1 | 1  8  1 |
| **URO (mmol/l)**  normal | 10 | 10 | 10 | 10 | 10 | 10 |
| **OSM (moms)**  Median  Mean  SD  Min  Max | 402.0  412.3  132.8  190  671 | 333.5  352.5  89.3  248  482 | 390.0  398.4  101.1  260  563 | 327.0  337.6  79.67  232  455 | 311.5  302.6  59.35  236  394 | 405.9  430.9 *  104.8  288  638 |

* confidence interval of SES to control does not include the zero value.

NOTE: individual significances (based on 95% confidence intervals of SES) for body weight, feed consumption and feed efficiency week-by-week have not been calculated
