# Supplementary material for: Variability of control data and relevance of observed group differences in five oral toxicity studies with genetically modified maize MON810 in rats
Source: Arch Toxicol. 2016 Oct 11;91(4):1977–2006. doi: 10.1007/s00204-016-1857-x (PMC5364247; doi:10.1007/s00204-016-1857-x)
Supplement: Supplementary file 3 — Supplementary material 3 (DOCX 17 kb) [file 204_2016_1857_MOESM3_ESM.docx]

**ESM-Table 3:** Histological findings in male and female Wistar Han RCC rats in the feeding trials D and E

| **Trial D** | | | |
| --- | --- | --- | --- |
| **Organ** | **Histological finding** | **33% isogenic non-GM maize** | **33% MON810 maize** |
| *Males*  heart  pancreas  prostate  small intestine  *Females*  small intestine | focal fibrosis, serous cyst on the atrioventricular valve  nodular hyperplasia  focal mononuclear infiltration  calcified epithelioid granuloma  calcified epithelioid granuloma | 0/10  0/10  2/10  0/10  0/10 | 1/10  1/10  1/10  1/10  3/10 |
|  | | | |
| **Trial E** | | | |
| **Organ** | **Histological finding** | **33% isogenic non-GM maize** | **33% MON810 maize** |
| *Males*  heart  prostate  small intestine  *Females*  ovarium  small intestine | necrotic nodule, mononuclear cell infiltration  focal mononuclear infiltration  calcified epithelioid granuloma  cyst  calcified epithelioid granuloma | 1/10  2/10  1/10  0/10  0/10 | 0/10  1/10  0/10  2/10  1/10 |
